# A repeated cross-sectional and longitudinal study of mental health and wellbeing during COVID-19 lockdowns in Victoria, Australia

**DOI:** 10.1186/s12889-022-14836-9

**Published:** 2022-12-27

**Authors:** Annemarie Wright, Alysha De Livera, Keun Hee Lee, Carl Higgs, Matthew Nicholson, Lisa Gibbs, Anthony Jorm

**Affiliations:** 1 Department of Health, Victorian State Government, Melbourne, Australia; 2 Melbourne School of Population and Global Health, The University of Melbourne, Melbourne, Australia; 3 Mathematical Sciences, School of Science, RMIT University, Melbourne, Australia; 4 Department of Mathematics and Statistics, La Trobe University, Melbourne, Australia; 5 College of Design and Social Context, RMIT University, Melbourne, Australia; 6 Monash University Malaysia, Kuala Lumpur, Malaysia

**Keywords:** COVID-19, Pandemic, Social connection, Mental health, Psychological distress, Life satisfaction

## Abstract

**Background:**

Population surveys across the world have examined the impact of the COVID-19 pandemic on mental health. However, few have simultaneously examined independent cross-sectional data with longitudinal data, each of which have different strengths and weaknesses and facilitate the investigation of distinct research questions. This study aimed to investigate psychological distress and life satisfaction during the first and second lockdowns in the state of Victoria, Australia, and the social factors that may be affected by lockdowns and could affect mental health.

**Methods:**

The VicHealth Victorian Coronavirus Wellbeing Impact Study included two 20-min opt-in online panel surveys conducted in May and September 2020 in Victoria, each with a sample of 2000 adults aged 18 + . A two-part study design was used: a repeated cross-sectional study of respondents who participated in Survey One and Survey Two, followed by a longitudinal nested cohort study. The primary exposures were social solidarity, social connectedness and staying connected with family and friends. Using logistic regression modelling, we explored the associations between our exposures and primary outcomes of psychological distress and life satisfaction with and without adjustment for covariates, both cross-sectionally and longitudinally. The results from the multivariable models were summarised using adjusted Odds Ratios (aOR), 95% Confidence Intervals (CI).

**Results:**

Cross-sectional results indicated that the percentage of participants with low life satisfaction was significantly higher in the second survey sample (53%) compared to the first (47%). The percentage of participants with high psychological distress was higher but not significantly different between the two survey samples (14% first survey vs 16% second survey). Longitudinal study results indicated that lower social connectedness was significantly associated with higher psychological distress (aOR:3.3; 95% CI: 1.3–8.4) and lower life satisfaction (aOR:0.2; 95% CI: 0.1–0.4). Younger adults had higher psychological distress compared to older adults (aOR:6.8; 95% CI:1.5–31.1). Unemployment at the time of the first survey was significantly associated with lower life satisfaction at the second survey (aOR:0.5; 95% CI: 0.3–0.9).

**Conclusion:**

This study supports the findings of other international studies. It also highlights the need to promote increased social connection and maintain it at times of isolation and separation, particularly amongst younger adults.

**Supplementary Information:**

The online version contains supplementary material available at 10.1186/s12889-022-14836-9.

## Introduction

The COVID-19 global pandemic is ongoing, caused by the SARS-CoV-2 virus. The first outbreak of infection was in China in December 2019, which was followed by spread to many other countries. The first case was detected in Australia on 25th January 2020. This case was in Melbourne, the capital of the state of Victoria. In the absence of vaccines at the time, the Victorian government attempted to control the spread of the virus using lockdowns, beginning on the 30^th^ March 2020. Victoria, and the city of Melbourne in particular, was the region of Australia most impacted by the pandemic and by series of lockdowns, which are summarized in Table 1. As in the rest of the world, the initial concern about the pandemic was the effect on mortality and hospitalizations. However, as the pandemic progressed, the potential effects on mental health have also been recognized [1]. Given the strong evidence for increased psychological distress and mental disorders after disasters [2], the prevalence of mental health problems was expected to rise due to infection and fear of infection, as well as the impacts of lockdowns, including social isolation, loss of employment and income, and intensification of pressures on families.

**Table 1:** Chronology of COVID-19 Restrictions in Victoria Up Until the Second Survey

Dates	Events and restrictions
25 Jan 2020	First COVID-19 case in Victoria (Melbourne)
30 March – 12 May 2020	Lockdown 1. This covered the whole of Victoria. People could only leave home for 4 reasons—food and supplies, exercise, medical care, work and education (if necessary). Non-essential businesses and schools were closed. People were required to work from home where possible. Gatherings of more than 2 people were banned (except for immediate household or for work or education purposes)
13 May – 8 July 2020	Restrictions were progressively eased across Victoria, but lockdowns were re-imposed on some areas of Melbourne that had new infections
9 July – 27 October 2020	Lockdown 2. This initially covered Melbourne and one shire adjacent to Melbourne, but restrictions were later imposed on regional Victoria from 4 August 2020. A night-time curfew was added for Melbourne from 2 August 2020

Sources: [3, 4]

There is now an extensive international literature exploring the mental health consequences of the pandemic. Early studies involved cross-sectional comparisons of the prevalence of symptoms of distress, anxiety and depression during the early months of the pandemic (March–April 2020) compared to pre-pandemic data; they found that the prevalence of symptoms was higher than pre-pandemic [5, 6]. Subsequent studies used stronger longitudinal designs and repeated cross-sectional surveys of representative samples. Systematic reviews and meta-analyses of these studies found that symptoms of distress, anxiety and depression increased during the early months (March–April) of the pandemic in 2020 but returned to pre-pandemic levels by the middle of that year [7, 8].

While longitudinal studies with frequent waves have been proposed as the best methodology for investigating pandemic-related mental health impacts [9], survivorship bias has been raised as a limitation of these studies. Czeisler et al. [10] analysed a 4-wave longitudinal study from the USA and found that people who participated in only 1 or 2 waves had a higher prevalence of depression and anxiety symptoms. This selective pattern of retention could lead to an overly optimistic interpretation of mental health over time. Czeisler et al. [10] therefore recommended the simultaneous use of independent cross-sectional data along with longitudinal data, as these approaches have different strengths and weaknesses, and facilitate the investigation of distinct research questions.

Most studies of the impact of the pandemic have assessed symptoms of mental disorders. However, Keyes [11] argued that a complete assessment of mental health should take account of a dimension of positive mental health as well as symptoms. Relevant to this two-dimensional concept, there have also been some studies on the effects of the pandemic on positive mental health, including subjective well-being, life satisfaction and positive affect. There has been less research carried out on positive mental health than on symptoms, but one systematic review and meta-analysis of studies up to June 2020 found no significant effect of COVID-19 pandemic lockdowns on measures of life satisfaction, positive affect, well-being and quality of life [12].

In addition to research examining changes in mental health measures, there have been studies of factors that may impact any changes found. A particular focus of pandemic studies has been social isolation and loneliness, as these are potentially adversely affected during lockdowns. While there was some evidence of an increase in these factors during the early phases of the pandemic [13], other systematic reviews found a lack of effect [7, 12]. One explanation of these findings is that there was an initial impact of restrictions and lockdowns on social relationships, but people soon adapted by finding alternative online means of social contact [12, 13] and that the shared experience of the pandemic may have increased social cohesion [12]. Studies from other types of disasters have also indicated that social factors such as access to social support and sense of social solidarity are associated with more positive mental health outcomes [14]. However, it was also shown that these factors can deteriorate over time.

The above findings mainly come from studies in Europe and North America and may not be generalizable to all countries. We focus on the studies with stronger methodologies involving more systematic and representative samples.

From early in the pandemic, the Australian Bureau of Statistics (ABS) has monitored the health and social impacts [15]. It found that symptoms of psychological distress increased in April 2020 when the nation was first affected by lockdowns, declined to pre-pandemic levels by June as restrictions eased, and then increased again in August when there was another wave of lockdowns. Another source of longitudinal data has come from the Life in Australia Panel [16]. This study found that the prevalence of severe psychological distress rose in April 2020 compared to pre-pandemic, but then fell in May. It worsened again from May to August, mainly due to the effects of a second lockdown in the state of Victoria. This impact subsequently declined, with Victoria showing little difference from the rest of the country by November 2020. A third longitudinal panel study is the Australian National COVID-19 Mental Health and Risk Communication Survey, which involved fortnightly on-line surveys from March to June 2020 [17]. This study found that any changes in depression and anxiety symptoms were generally transitory. The study also found that while suicidal ideation was high, it did not vary over time [18]. Later work from this study reported greater psychological distress in parents and caregivers who were home schooling, but no effect on wellbeing [19]. Overall, the Australian data show a worsening of mental health which was associated with lockdowns, but this effect diminished over time.

Australian studies of mental health outcomes from other types of disaster are also of relevance, including an epidemiological study showing increased risk of psychiatric disorders post disaster [20], cross-sectional analyses demonstrating a complex relationship between social networks and individual mental health post bushfires [21], and longitudinal analyses indicating moderate involvement in community groups can be a protective factor for mental health following bushfire exposure [22].

In the present study, we report new repeated cross-sectional and longitudinal data from the Australian state of Victoria, which was the region of Australia most affected by pandemic lockdowns. The capital city of Melbourne was particularly affected and by 2021 was one of the most locked-down cities in the world [23]. Consistent with Keyes’ [11] two-factor concept of mental health, the study aimed to assess both symptoms of psychological distress and life satisfaction. We aimed to investigate cross-sectional factors associated with higher psychological distress and life satisfaction at each survey, and also social connection factors associated with longitudinal changes between the two surveys.

## Method

This paper builds on the descriptive findings of the Victorian Coronavirus Wellbeing Impact Study surveys [24], which showed significantly higher rates of low-medium life satisfaction over time and a non-significant trend towards higher rates of psychological distress over time.

### Study design

The current analysis has a two-part study design: a repeated cross-sectional study of respondents who participated in Survey One and Survey Two, followed by a longitudinal nested cohort study of these same respondents. The surveys were undertaken by the Victorian Health Promotion Foundation (VicHealth), a state-based government agency in Victoria, Australia, with a remit to promote health and prevent illness.

The VicHealth Coronavirus Victorian Wellbeing Impact Surveys of Victorian residents aged 18 years and over were conducted via an opt-in ‘research only’ online panel (i.e. non-probability panel). The surveys were designed to track the impact of the pandemic and associated lockdowns on a range of behavioural and attitudinal health risk factors during the first two waves of the pandemic in Victoria, from March to June in 2020 and from July through to October 2020.

Survey One commenced on 31 May 2020 and concluded 8 June 2020. The total achieved sample size was 2,000. Survey Two, which was conducted during the second pandemic wave, commenced on 10 September 2020 and concluded on 21 September 2020. Survey Two included 1,008 respondents who were re-contacted from Survey One and 992 ‘new’ respondents (i.e. those who did not complete Survey One), to boost the total sample size to 2,000.

### Participants

The opt-in online panel used for both surveys was LiveTribe, a research-only panel operated and managed by i-Link Research https://www.i-linkresearch.com/. LiveTribe panellists are recruited via print media, online marketing initiatives, direct mail, social media platforms, affiliate partnerships, personal invitations and a range of other ad-hoc initiatives. For Survey One and Two the sample size of 2,000 was chosen as it provides a reasonable margin of error for the purposes of estimating population level results within an expedited fieldwork turnaround period.

### Survey questionnaire

The 20-min survey questionnaire (see Supplementary file 1) covered a range of health and wellbeing factors, including life satisfaction, subjective wellbeing, psychological distress and social connection, as well as socio-demographics. The selection of health and wellbeing indicators for the questionnaire was guided by several key principles previously used in population surveys conducted by VicHealth [25]. These principles included: sensitivity to change across person, place and time; strong psychometric properties; being amenable to action at a range of jurisdiction levels including local government; non-duplication of other Victorian population surveys; and brief questions that could be feasibly used in local program evaluations, thus allowing the population measure to act as a comparator for local evaluations.

Different question styles were used to minimise respondent fatigue and enhance engagement with the survey, such as Likert scales, closed-ended questions and open-ended questions. Current guidelines were followed to ensure questions were as user-friendly as possible for respondents, regardless of the device being used to access the survey (for example, mobile phones, tablets, desktops or laptops) [26]. Respondents were also asked to provide their nearest cross-street to enable the application of geo-codes to participants’ identification codes and their question responses.

No formal pilot testing of the survey was undertaken. However, a soft launch was undertaken to confirm the integrity of the questionnaire. The soft launch involved inviting a small number of participants to complete the survey, with the aim of securing approximately 20 completed surveys. The data from these surveys were carefully checked against the Microsoft Word version of the survey to ensure the survey was error free and had been scripted as expected. No errors were detected as a result of this process and the survey was launched in full without any amendment. The average completion time of the questionnaire was 20 min.

### Measures

Our primary outcomes were psychological distress and life satisfaction, and the primary exposures were feeling connected with others, staying connected with family and friends, and social solidarity.

‘Psychological distress’ was measured using the Kessler Psychological Distress Scale-6 (K6) which has excellent internal consistency reliability (Cronbach's alpha = 0.89) [27]. The K6 is a combined score across 6 areas of psychological distress; each person can score a minimum of 6 and maximum of 30. Scores of 19 or more are classified as probable serious mental illness, while scores of 6 to 18 are classified as no probable serious mental illness. Null responses to 2 or more of the 6 statements were excluded from the mean calculation, with adjustments made for those who gave a null response to 1 statement.

‘Life satisfaction’ [28] was derived from a rating of satisfaction with life as a whole using a scale of 0 to 10 where 0 is completely dissatisfied and 10 is completely satisfied. The measure correlates highly with the Personal Wellbeing Index measure of life satisfaction (*r* = 0.79) [29]. Low to medium life satisfaction was defined as a score between 0 and 6 out of 10. Null responses were excluded from mean calculation.

The measure of ‘Feeling connected with others’ [30] uses a six item Likert scale to assess level of agreement with the statement ‘I feel connected with others’. The measure has a high positive correlation (+ 0.79) with the Social Connectedness Revised Scale and a high negative correlation (-0.69) with the UCLA Loneliness Scale [30]. For analysis, responses were divided into the percentage of people who selected strongly disagree, disagree, mildly disagree, mildly agree, and those who selected agree or strongly agree.

‘Staying connected with family and friends’ was a question developed for the survey and to the best of our knowledge it is the first time that this measure has been used. It measures the extent to which people find it easy or hard to stay connected with those close to them. For analysis, the five-point Likert scale was coded as the percentage of people who reported it was 1. very easy or easy; 2. neither or 3. hard or very hard.

The **‘**Social solidarity’ measure [31] was designed to determine how close people feel with their communities using a combined score across six questions and has good reliability across samples (Cronbach’s alpha ranging from 0.78 to 0.89). These questions ask respondents whether they agree with statements regarding their connection with their local community. Responses for all six questions were assigned the following values: Strongly disagree = 1, Disagree = 2, Neither agree nor disagree = 3, Agree = 4, Strongly agree = 5. Any respondent providing a ‘don’t know’ or ‘prefer not to answer’ response to any of the six questions was excluded from the analysis. The final score was calculated by summing the values of the six categories out of a maximum of 30 and minimum of six.

In addition to the above, the variables age, gender, disability, income, the main activity in September, region, and household composition were used as covariates in the longitudinal analysis. The covariates used for each analysis are shown in the corresponding tables.

### Ethics

Ethics approval for Survey One was provided by the Australian National University Human Research Ethics Committee (2020/264) on 20 May 2020. Ethics approval for Survey Two was provided by the Australian National University Human Research Ethics Committee (2020/540) on 8 September 2020. All methods were carried out in accordance with relevant guidelines and regulations. Online written informed consent was obtained from study participants as this survey was carried out as an online survey.

### Statistical analysis

Data were analysed using Stata, V.17 [32] and R software [33]. Categorical data were summarised using frequency with percentages, continuous data using means with standard deviations, and skewed data using medians with interquartile ranges (25th –75th centile). Our primary outcomes, exposures and covariates are described in the Measures section. To assess differences in dependent means and proportions paired sample t-tests and McNemar’s tests were used respectively. Using logistic regression modelling, we explored the associations between our exposures and primary outcomes of psychological distress and life satisfaction with and without adjustment for covariates. The results from the multivariable models were summarised using adjusted Odds Ratios (aOR), 95% Confidence Intervals (CI) and p-values. To identify confounding variables for the regression analyses, we used information from previous literature and Directed Acyclic Graphs (DAG) [34, 35]. For each outcome, we conducted three separate analyses: (i) a cross-sectional analysis at the first lockdown, (ii) a cross-sectional analysis at the second lockdown, and (iii) a longitudinal analysis exploring the change in outcome measures between the two lockdowns. We tested for exposure-outcome associations that may have been modified by other covariates using interaction terms and likelihood ratio tests, and used measures of fit for logistic regression [36] to test model assumptions. We used complete-case analysis (i.e., analysis restricted to participants with complete data), and investigated the baseline first-lockdown characteristics of those missing and not-missing at the second lockdown using summary statistics.

## Results

### Cohort characteristics

The cohort characteristics for the 1008 participants who completed the survey at both lockdowns are shown in Table 2. Table 2 indicates that this sample consists of a higher proportion of female participants (56%) compared to male, a higher proportion living in Melbourne (77%) compared to regional areas, and a higher proportion reporting not having a disability (75%). Approximately 56% were employed in February 2020 and 55% employed in September 2020, while the rest consisted of retired, student and other in February and September 2020. Approximately 32% of the household composition consisted of couples with children, and the rest consisted of couple living alone, person living alone, single parent with children and other. Compared to the first lockdown, at the second lockdown participants had lower life satisfaction, felt less connected with others, had a harder time connecting with family and friends, and had lower social solidarity.

**Table 2:** Cohort characteristics at the first lockdown & the second lockdown of the participants who completed both surveys

	First lockdown	Second lockdown
	*N* = 1,008 N (%) or mean (SD)^a *^	*N* = 1,008 N (%) or mean (SD)^a*^
Combined score of social solidarity (Aggregated score from 6 to 30)	21.53 (4.36)	20.88 (4.72)
I feel connected with others		
Agree	408 (40.5%)	317 (31.4%)
Mildly	328 (32.5%)	353 (35.0%)
Disagree	221 (21.9%)	288 (28.6%)
Missing	51 (5.1%)	50 (5.0%)
Stay connected with family and friends		
Easy	393 (39.0%)	280 (27.8%)
Neither	313 (31.1%)	277 (27.5%)
Hard	272 (27.0%)	424 (42.1%)
Missing	30 (3.0%)	27 (2.7%)
Life satisfaction		
Low/Medium (Rated 0 to 6)	463 (45.9%)	548 (54.4%)
High (Rated 7 or 10)	526 (52.2%)	437 (43.4%)
Missing	19 (1.9%)	23 (2.3%)
Psychological distress		
No probable mental health issue	848 (84.1%)	835 (82.8%)
High Psychological Distress	124 (12.3%)	142 (14.1%)
Missing	36 (3.6%)	31 (3.1%)
Age		
65 + years	193 (19.1%)	197 (19.5%)
55 – 64 years	234 (23.2%)	239 (23.7%)
45 – 54 years	224 (22.2%)	230 (22.8%)
35 – 44 years	179 (17.8%)	180 (17.9%)
18 – 34 years	178 (17.7%)	162 (16.1%)
Gender		
Male or Non-binary	439 (43.6%)	439 (43.6%)
Female	568 (56.3%)	568 (56.3%)
Missing	1 (0.1%)	1 (0.1%)
Disability		
No	754 (74.8%)	747 (74.1%)
Yes	213 (21.1%)	203 (20.1%)
Missing	41 (4.1%)	58 (5.8%)
Income		
$100,000 or more	263 (26.1%)	259 (25.7%)
$60,000 – $99,999	215 (21.3%)	214 (21.2%)
$40,000 – $59,999	147 (14.6%)	158 (15.7%)
Under $40,000	245 (24.3%)	245 (24.3%)
Missing	138 (13.7%)	132 (13.1%)
Main activity in February & September 2020	(February)	(September)
Employed	566 (56.2%)	553 (54.9%)
Unemployed	176 (17.5%)	200 (19.8%)
Retired	210 (20.8%)	218 (21.6%)
Other	40 (4.0%)	28 (2.8%)
Missing	16 (1.6%)	9 (0.9%)
Region		
Other	233 (23.1%)	233 (23.1%)
Melbourne	775 (76.9%)	775 (76.9%)
Household Composition		
Couple living alone	291 (28.9%)	291 (28.9%)
Person living alone	220 (21.8%)	220 (21.8%)
Couple with children	322 (31.9%)	319 (31.6%)
Single parent with children	66 (6.5%)	66 (6.5%)
Other	88 (8.7%)	90 (8.9%)
Missing	21 (2.1%)	22 (2.2%)

*N* Number, *SD* Standard deviation

^*^Statistically significant differences between the two lockdowns are highlighted in grey background using an arbitrary p-value cut-off of 0.05 [37]. Paired t-tests and McNemar’s tests were used for comparing means and proportions respectively. Data consists of the participants who completed both surveys

^a^ Unless otherwise stated

The cohort characteristics are summarised separately at the first and the second lockdowns in Supplementary File 2 Table S1. The participants’ characteristics at the first lockdown for those who were missing and not missing at the second lockdown are shown in Supplementary File 2 Table 1. Supplementary File 2 Table S2 shows that those who were missing at the second lockdown compared to those who were not missing, had higher psychological distress, and were more likely to be male, live in Melbourne, and be employed in February 2020.

### Cross-sectional results at the first lockdown

Cross-sectional results from logistic regression modelling at the first lockdown with psychological distress and life satisfaction as outcomes are shown in Table 3.

**Table 3:** Results from multivariable logistic regression modelling with psychological distress and life satisfaction as outcome at the first lockdown*

Variable	Psychological Distress^a^ aOR, (95% CI), *p*-value	Life Satisfaction^b^ aOR, (95% CI), *p*-value
Social Solidarity	0.96 (0.92, 1.00), 0.05	1.09 (1.06, 1.12), < 0.001
Feel connected with others		
Agree	**-**	**-**
Mildly	1.37 (0.86, 2.17), 0.19	0.58 (0.44, 0.77), < 0.001
Disagree	3.84 (2.37, 6.22), < 0.001	0.28 (0.20, 0.40), < 0.001
Stay connected with family and friends		
Easy	**-**	**-**
Neither	0.94 (0.58, 1.53), 0.81	0.66 (0.49, 0.89), 0.01
Hard	1.86 (1.21, 2.86), < 0.001	0.45 (0.33, 0.62), < 0.001
Age		
65 + years	**-**	**-**
55—64 years	1.67 (0.67, 4.20), 0.27	0.71 (0.43, 1.17), 0.18
45—54 years	2.93 (1.11, 7.75), 0.03	0.55 (0.32, 0.95), 0.03
35—44 years	3.68 (1.36, 9.93), 0.01	0.48 (0.27, 0.86), 0.01
18—34 years	8.25 (3.25, 20.97), < 0.001	0.45 (0.27, 0.78), < 0.001
Gender		
Male or Non-binary	**-**	**-**
Female	1.11 (0.77, 1.60), 0.59	0.88 (0.68, 1.13), 0.32
Disability		
No	**-**	**-**
Yes	4.20 (2.70, 6.55), < 0.001	0.59 (0.42, 0.82), < 0.001
Income		
$100,000 or more	**-**	**-**
$60,000—$99,999	1.85 (1.13, 3.03), 0.01	0.84 (0.60, 1.17), 0.30
$40,000—$59,999	1.86 (1.04, 3.33), 0.04	0.60 (0.41, 0.89), 0.01
Under $40,000	1.85 (1.01, 3.41), 0.05	0.48 (0.32, 0.72), < 0.001
Main activity in Feb		
Employed	**-**	**-**
Unemployed	0.60 (0.36, 1.02), 0.06	0.83 (0.57, 1.22), 0.35
Retired	0.64 (0.28, 1.51), 0.31	1.09 (0.67, 1.79), 0.73
Other	0.73 (0.32, 1.63), 0.44	0.64 (0.35, 1.14), 0.13
Region		
Other	**-**	**-**
Melbourne	1.91 (1.20, 3.04), 0.01	1.04 (0.77, 1.40), 0.81
Household Composition		
Couple living alone	**-**	**-**
Person living alone	1.13 (0.65, 1.95), 0.67	0.85 (0.58, 1.24), 0.39
Couple with children	0.90 (0.54, 1.49), 0.68	0.96 (0.68, 1.35), 0.81
Single parent with children	0.72 (0.34, 1.53), 0.40	0.67 (0.39, 1.16), 0.15
Other	0.38 (0.19, 0.77), 0.01	0.89 (0.57, 1.41), 0.63

Results using cross-sectional data are presented; Statistically significant associations are highlighted in grey background using an arbitrary *p*-value cut-off of 0.05 [37].Unadjusted modelling is shown in Supplementary File 2, Table S3

*aOR* Adjusted Odds Ratio, *CI* Confidence Interval

^*^ Models are adjusted for the covariates shown in the table

^a^ N_1_ = 1287

^b^ N_2_ = 1287

At the first lockdown, participants who found it hard to stay connected with others had 1.86 (95% CI: 1.2–2.9) times higher odds of psychological distress compared to those who found it easy to stay connected after adjusting for covariates. Participants who did not feel connected with others had 3.8 (95% CI: 2.4–6.2) times higher odds of psychological distress than those who felt connected. Higher social solidarity was associated with lower odds of psychological distress (Odds Ratio, OR = 0.96, 95% CI: 0.9–1.0). Melbourne residents had higher odds of psychological distress compared to those who lived outside Melbourne. Psychological distress was higher for younger participants. Participants who reported a disability had higher odds of psychological distress compared to those who did not report disability. In addition, lower income was associated with higher psychological distress.

A similar range of variables were found to be associated with life satisfaction at the first lockdown (Table 3). Participants who did not feel connected with others had 0.3 (95% CI: 0.2–0.4) times lower odds of life satisfaction than those who felt connected after accounting for covariates. Those who found it hard to stay connected with others had 0.5 (95% CI: 0.3–0.6) times lower odds of life satisfaction compared to those who found it easy to stay connected. Higher social solidarity was associated with higher odds of life satisfaction (OR = 1.09, 95% CI: 1.06–1.12). Life satisfaction was lower for younger participants. Participants who reported disability had lower odds of life satisfaction compared to those who did not report disability. Lower income was associated with lower life satisfaction.

### Cross-sectional results at the second lockdown

Cross-sectional results at the second lockdown for the outcome variables psychological distress and life satisfaction are shown in Table 4.

**Table 4:** Results from multivariable logistic regression modelling with psychological distress and life satisfaction as outcome at the second lockdown*

Variable	Psychological Distress^a^ aOR, (95% CI), *p*-value	Life Satisfaction^b^ aOR, (95% CI), *p*-value
Social Solidarity	0.94 (0.91, 0.98), < 0.001	1.06 (1.03, 1.09), < 0.001
Feel connected with others		
Agree	-	-
Mildly	0.70 (0.44, 1.11), 0.13	0.46 (0.35, 0.62), < 0.001
Disagree	2.57 (1.65, 4.00), < 0.001	0.22 (0.15, 0.32), < 0.001
Stay connected with family and friends		
Easy	-	-
Neither	0.60 (0.37, 0.99), 0.05	0.50 (0.36, 0.70), < 0.001
Hard	1.42 (0.94, 2.14), 0.10	0.29 (0.21, 0.40), < 0.001
Age		
65 + years	-	-
55—64 years	3.12 (1.55, 6.29), < 0.001	0.93 (0.59, 1.47), 0.77
45—54 years	3.54 (1.59, 7.85), < 0.001	0.91 (0.54, 1.54), 0.72
35—44 years	3.91 (1.70, 9.01), < 0.001	0.57 (0.33, 0.99), 0.05
18—34 years	7.69 (3.50, 16.87), < 0.001	0.66 (0.40, 1.10), 0.11
Gender		
Male or Non-binary	-	-
Female	1.20 (0.86, 1.67), 0.29	0.79 (0.61, 1.02), 0.07
Disability		
No	-	-
Yes	2.29 (1.50, 3.50), < 0.001	0.52 (0.37, 0.73), < 0.001
Income		
$100,000 or more	-	-
$60,000—$99,999	1.01 (0.65, 1.57), 0.97	0.62 (0.44, 0.86), < 0.001
$40,000—$59,999	1.04 (0.62, 1.73), 0.89	0.93 (0.63, 1.38), 0.72
Under $40,000	1.02 (0.58, 1.77), 0.96	0.93 (0.61, 1.42), 0.74
Main activity in Sep		
Employed	-	-
Unemployed	1.08 (0.68, 1.72), 0.75	0.53 (0.36, 0.79), < 0.001
Retired	1.11 (0.55, 2.20), 0.78	1.07 (0.67, 1.70), 0.78
Other	1.46 (0.67, 3.21), 0.34	0.94 (0.47, 1.89), 0.86
Region		
Other	-	-
Melbourne	1.60 (1.03, 2.47), 0.04	0.92 (0.68, 1.24), 0.59
Household Composition		
Couple living alone	-	-
Person living alone	1.08 (0.67, 1.75), 0.75	0.84 (0.58, 1.20), 0.34
Couple with children	1.02 (0.64, 1.61), 0.94	1.18 (0.84, 1.68), 0.34
Single parent with children	1.15 (0.58, 2.29), 0.69	0.62 (0.34, 1.11), 0.11
Other	0.93 (0.49, 1.77), 0.83	0.50 (0.30, 0.84), 0.01

Results using cross-sectional data are presented; Statistically significant associations are highlighted in grey background using an arbitrary p-value cut-off of 0.05 [37]. Unadjusted modelling is shown in Supplementary File 2, Table S4

^*^ Models are adjusted for the covariates shown in the table

*aOR* Adjusted Odds Ratio, *CI* Confidence Interval

^a^ N_1_ = 1377

^b^ N2 = 1382

The cross-sectional results at the second lockdown were comparable to the first lockdown. Participants who did not feel connected with others had 2.6 (95% CI: 1.7–4.0) times higher odds of psychological distress than those who felt connected after adjusting for covariates. Higher social solidarity was associated with lower odds of psychological distress (OR = 0.94, 95% CI: 0.9–1.0). Psychological distress was higher for younger participants. Participants who reported a disability had higher odds of psychological distress compared to those who did not report disability. Melbourne residents had higher odds of psychological distress compared to those who lived outside Melbourne.

Participants who did not feel connected with others had lower life satisfaction compared to those who felt connected (OR = 0.2, 95% CI: 0.2–0.3). The participants who found it hard to stay connected with others had 0.3 (95% CI: 0.2–0.4) times lower odds of life satisfaction compared to those who found it easy to stay connected. Higher social solidarity was associated with higher odds of life satisfaction (OR = 1.06, 95% CI: 1.03–1.09). Participants who reported a disability had lower odds of life satisfaction compared to those who did not report disability. Participants who were unemployed in the September had lower odds of life satisfaction compared to those who were employed.

### Longitudinal results

Longitudinal results for psychological distress and life satisfaction are presented in Table 5.

**Table 5:** Longitudinal associations of change in psychological distress between the first and the second lockdowns^*^

Variable	Psychological Distress^**a**^ aOR, (95% CI), *p*-value	Life Satisfaction^**b**^ aOR, (95% CI), *p*-value
Social Solidarity at the first lockdown	1.01 (0.92, 1.11), 0.82	1.05 (0.97, 1.14), 0.22
Social Solidarity at the second lockdown	0.96 (0.87, 1.05), 0.35	1.00 (0.93, 1.08), 0.99
Feel connected with others at the first lockdown		
Agree	-	-
Mildly	1.47 (0.68, 3.18), 0.33	0.83 (0.52, 1.34), 0.44
Disagree	1.08 (0.43, 2.67), 0.87	1.35 (0.69, 2.65), 0.39
Feel connected with others at the second lockdown		
Agree	-	-
Mildly	0.47 (0.18, 1.23), 0.12	0.53 (0.32, 0.89), 0.02
Disagree	3.25 (1.26, 8.43), 0.02	0.18 (0.09, 0.35), < 0.001
Stay connected with family and friends at the first lockdown		
Easy	-	-
Neither	0.75 (0.32, 1.73), 0.50	0.81 (0.49, 1.34), 0.41
Hard	0.84 (0.37, 1.88), 0.67	0.63 (0.36, 1.13), 0.12
Stay connected with family and friends at the second lockdown		
Easy	-	-
Neither	1.34 (0.52, 3.47), 0.55	0.55 (0.31, 0.96), 0.03
Hard	1.46 (0.59, 3.61), 0.42	0.36 (0.20, 0.64), < 0.001
Age		
65 + years	-	-
55—64 years	1.80 (0.48, 6.82), 0.39	1.07 (0.51, 2.23), 0.86
45—54 years	4.05 (0.88, 18.57), 0.07	1.03 (0.43, 2.48), 0.94
35—44 years	3.20 (0.63, 16.22), 0.16	0.70 (0.27, 1.77), 0.45
18—34 years	6.77 (1.47, 31.07), 0.01	0.51 (0.21, 1.23), 0.13
Gender		
Male or Non-binary	-	-
Female	1.27 (0.67, 2.43), 0.46	0.73 (0.47, 1.13), 0.16
Disability		
No	-	-
Yes	1.34 (0.60, 3.00), 0.47	0.59 (0.33, 1.06), 0.08
Income		
$100,000 or more	-	-
$60,000—$99,999	0.44 (0.18, 1.10), 0.08	0.57 (0.32, 1.02), 0.06
$40,000—$59,999	0.55 (0.22, 1.42), 0.22	1.11 (0.57, 2.16), 0.75
Under $40,000	0.74 (0.26, 2.08), 0.57	0.99 (0.48, 2.06), 0.98
Main activity in September		
Employed	-	-
Unemployed	2.22 (0.95, 5.21), 0.07	0.49 (0.25, 0.95), 0.04
Retired	2.78 (0.76, 10.21), 0.12	0.74 (0.34, 1.60), 0.44
Other	0.92 (0.08, 10.60), 0.95	0.28 (0.06, 1.38), 0.12
Region		
Other	-	-
Melbourne	1.21 (0.57, 2.57), 0.62	1.06 (0.65, 1.73), 0.81
Household Composition		
Couple living alone	-	-
Person living alone	0.83 (0.31, 2.20), 0.70	1.04 (0.55, 1.99), 0.89
Couple with children	1.37 (0.56, 3.33), 0.49	1.51 (0.83, 2.77), 0.18
Single parent with children	1.25 (0.33, 4.69), 0.74	0.70 (0.27, 1.80), 0.45
Other	1.35 (0.39, 4.71), 0.63	0.72 (0.29, 1.78), 0.48
Psychological Distress at the first lockdown	17.93 (8.03, 40.02), < 0.001	-
Life Satisfaction at the first lockdown	-	5.04 (3.22, 7.91), < 0.001

Results using longitudinal data are presented; Statistically significant associations are highlighted in grey background using an arbitrary p-value cut-off of 0.05 [37]. Unadjusted modelling is shown in Supplementary File 2, Table S5

*aOR* Adjusted Odds Ratio, *CI* Confidence Interval

^*^Model adjusted for the covariates shown in the table

^a^ N_1_ = 591

^b^ N_2_ = 592

The results showed that participants who did not feel connected with others at the second lockdown had 3.3 (95% CI: 1.3–8.4) times higher odds of psychological distress at the second lockdown after controlling for their psychological distress status at the first lockdown and other covariates (Table 5). The younger participants aged less than 35 years had higher odds of psychological distress compared to participants over 65 years of age. Participants who did not feel connected with others and found it hard to stay connected with the family and friends at the second lockdown had lower life satisfaction at the second lockdown—with OR = 0.2 (95% CI: 0.1–0.4) and OR = 0.4 (95% CI: 0.2–0.6) respectively—after accounting for their life satisfaction at the first lockdown and other covariates (Table 5). No significant associations between social connection (feeling connected, staying connected, social solidarity) at the first lockdown and psychological distress or life satisfaction at the second lockdown were found after controlling for covariates.

## Discussion

This study aimed to investigate psychological distress and life satisfaction during the first and second lockdowns in the state of Victoria in 2020, as well as social factors that may be affected by lockdowns and which could affect mental health. The main finding was that adults aged 18 years and over who did not feel connected with others at the second lockdown had 3.3 times higher odds of psychological distress at the second lockdown, after controlling for their psychological distress status at the first lockdown and other covariates. This odds ratio is considered to be a small to medium effect size [38].

### Cross-sectional findings

Using life satisfaction as an indicator of positive mental health, we found that the percentage of participants with low life satisfaction was significantly higher in the second survey sample (53.1%) compared to the first survey sample (46.9%). Because the survey samples are not representative, these percentages could be biased figures as estimates for prevalence. However, a previous descriptive analysis that weighted the data by population characteristics found a similar significant change for prevalence of low life-satisfaction from 49 to 53% [24]. This result differs from a previous systematic review and meta-analysis of studies up to June 2020 which found no significant effect of COVID-19 pandemic lockdowns on life satisfaction. However, these studies examined the effects of single lockdowns, whilst the results of this study indicate that a second lockdown led to a decrease in life satisfaction.

By contrast, the percentage of participants with high psychological distress was not significantly different between the two survey samples, with high distress found in 14.3% in the first survey sample compared to 15.6% in the second survey sample. In comparison to the prevalence percentages reported previously where weighted data had been used, the prevalence percentages were higher, but again there was no change across surveys (16% vs 17%) [24]. These results are different to other studies that have found that psychological distress reduced after initially increasing during the early months (March–April 2020) of the pandemic [7, 8]. As the first survey was conducted in May–June 2020, it may be that this study did not capture the initial peak in psychological distress.

Whilst this study did not have a pre-COVID baseline, we can get some indication of the impact of lockdowns in general through examining the Melbourne region, which was subjected to greater restrictions than the rest of Victoria, particularly during the second lockdown. In the cross-sectional analyses from both lockdowns, residents of Melbourne had a higher level of psychological distress after adjusting for other factors, indicating that greater lockdown is likely to be a risk factor. These findings are consistent with studies internationally showing that lockdowns have a negative impact on symptoms of mental health problems [5, 6]. On the other hand, we found no effect of living in Melbourne compared to the rest of the state on life satisfaction, whereas there was a decrease in life satisfaction across the state as a whole from the first to the second lockdown.

When the social connection factors were examined, there was a higher proportion of participants in the second survey reporting not feeling connected to others and finding it hard to stay connected to family and friends. There was also a lower social solidarity mean score. These findings are consistent with the international literature that there was an increase in social isolation and loneliness in the early phase of the pandemic [13], but are not consistent with suggestions that the effect of social isolation diminished over time as people adapted by finding alternative online means of social contact [12, 13]. However, the findings are consistent with the broader literature on disasters showing a potential deterioration in social support and social solidarity over time following a critical incident [14].

### Longitudinal findings

The longitudinal analysis facilitated the investigation of factors associated with changes in life satisfaction and psychological distress from the first to the second lockdown.

Lower social connectedness was associated with worsening of both psychological distress and life satisfaction. Not feeling connected with others was associated with both mental health variables, while finding it hard to stay connected was specifically associated with lower life satisfaction. This is consistent with the research following the Black Saturday bushfires which showed the relationship between social networks and mental health post disaster [21] and identified connection to community groups as a protective factor [22]. These associations point to interventions targeting social connectedness as a potential response to future lockdowns.

While lower social connectedness was associated with both increases in psychological distress and decreases in life satisfaction, there were also some differential associations. A notable longitudinal finding was that younger adults had a greater increase in psychological distress across lockdowns. This association was found even after adjustment for social risk factors and indicates that young people should be a particular target for intervention to reduce the psychological impact of lockdowns. This has been shown in other post-disaster studies and may reflect lower maturity and life experience [39]. However, by contrast, the younger adult age group was not associated with greater declines in life satisfaction.

Unemployment at the time of the first survey was also associated with declines in life satisfaction. Although the association with psychological distress was not statistically significant at our pre-set significance level, there was a trend in the same direction. However, any negative impact on mental health in the second lockdown may have been ameliorated by changes to Australian government policy in March 2020, that came into full effect after the first lockdown. This policy increased welfare payments to people who were unemployed in an effort to stimulate the economy (the JobSeeker scheme). There was also a JobKeeper scheme to subsidize wages of people in employment where the pandemic was adversely affecting the business of their employer, which may have reduced distress associated with fear of unemployment.

In the longitudinal analysis, we found no effect of living in Melbourne on changes in either psychological distress or life satisfaction, indicating that any worsening of mental health across lockdowns was not greater in Melbourne, despite the greater restrictions on the city in the second lockdown. At the time of the second survey (10–21 September 2020), Melbourne had been in lockdown for two months and had been under curfew for one month, whereas the rest of Victoria had been in lockdown only for approximately one month. It may be that the difference in accumulated lockdown ‘dose’ between Melbourne and the rest of Victoria at the time of the second survey was not significant enough to show a difference in psychological distress and life satisfaction. Alternatively, given that the cross-sectional analysis did show a difference in Victorian regions, the lack of effect in the longitudinal analysis may be an indicator of survivorship bias, with those experiencing higher psychological distress not participating in the second survey, consistent with the limitations of longitudinal studies reported previously [10].

### Limitations

An important limitation is that the samples were taken from an opt-in online panel with complex recruitment strategies, which is unlikely to be representative. Some sub-groups are likely to be excluded by the survey method, such as people without digital access and/or without digital literacy or English literacy. While representativeness is not required for the longitudinal analysis of risk factors, it potentially affects prevalence estimates in the cross-sectional analyses. However, prevalence changes between surveys were similar when there was weighting of the sample by population characteristics [24], which adds to confidence in the findings.

In their longitudinal study of mental health during the pandemic, Czeisler et al. [10] found that there was a high dropout rate in people with worse mental health, which could lead to an overly optimistic interpretation of mental health over time. Our data are consistent with this finding, with the proportion of participants with high psychological distress in the first survey being greater for those who did not participate in the second survey compared to those who did (16% vs 12%). Thus, the proportion of people with high psychological distress is likely to be under-estimated at the second survey. However, we did not find a significant difference between these groups in the proportion of participants with low life satisfaction (48% vs 46%).

## Conclusion

The state of Victoria, and the city of Melbourne in particular, was the area of Australia most impacted by COVID lockdowns. The cross-sectional data collected in the first and second lockdowns, reported here, facilitated an investigation of the factors associated with higher psychological distress and life satisfaction, consistent with Keyes’ [11] two-continua model of mental health, while the longitudinal data enabled an exploration of the change between the two lockdowns. The data showed that lower social connectedness was associated with worsening psychological distress and life satisfaction, and that the young adults had a greater increase in psychological distress across the lockdowns.

The results in this study support and expand on findings of other international studies. They also indicate that in any future pandemics or lockdowns, interventions designed to maintain social connectedness may ameliorate the risk of decreased well-being and increased psychological distress. This is particularly important for younger adults. Routine promotion of involvement in community groups may be protective [22].

## Supplementary Information

**Additional file 1.** VicHealth Coronavirus Victorian Wellbeing Impact Survey Questionnaire.

**Additional file 2:** **Table S1.** Cohort characteristics at the first lockdown & the second lockdowns. **Table S2.** Baseline characteristics of the cohort with and without missing at the second lockdown. **Table S3.** Results from univariable logistic regression modelling with psychological distress and life satisfaction asoutcome at the first lockdown^**^. **Table S4.** Results from univariable logistic regression modelling with psychological distress and life satisfaction as outcome at the second lockdown^**^. **Table S5.** Longitudinal associations of change in psychological distress and life satisfaction between the first and the second lockdowns^**^.

## Data Availability

The de-identified dataset used and analysed during this current study is available from the corresponding author on reasonable request.

## Abbreviations

ABS: Australian Bureau of Statistics
CI: Confidence Interval
DAG: Directed Acyclic Graphs
K6: Kessler Psychological Distress Scale-6
OR: Odds Ratio
aOR: Adjusted Odds Ratio
SD: Standard Deviation
VicHealth: Victorian Health Promotion Foundation
