# Supplementary material for: A repeated cross-sectional and longitudinal study of mental health and wellbeing during COVID-19 lockdowns in Victoria, Australia
Source: BMC Public Health. 2022 Dec 27;22:2434. doi: 10.1186/s12889-022-14836-9 (PMC9793381; doi:10.1186/s12889-022-14836-9)
Supplement: Supplementary file 1 — Additional file 1. VicHealth Coronavirus Victorian Wellbeing Impact Survey Questionnaire. [file 12889_2022_14836_MOESM1_ESM.docx]

**Supplementary File 1**

**VicHealth Coronavirus Victorian Wellbeing Impact Survey Questionnaire**

**MODULE A: GENERAL WELLBEING**

*(ALL)

A1W Thinking about your own life and your personal circumstances, how satisfied are you with your life as a whole? Please use a scale from 0-10, where 0 is completely dissatisfied and 10 is completely satisfied.

*Please provide a response for the time during the current (August and September) coronavirus restrictions.*

|  | During the current *(August and September)* coronavirus restrictions |
| --- | --- |
| 0 – Completely dissatisfied |  |
| 1 |  |
| 2 |  |
| 3 |  |
| 4 |  |
| 5 |  |
| 6 |  |
| 7 |  |
| 8 |  |
| 9 |  |
| 10 – Completely satisfied |  |
| 98. Not sure |  |
| 99. Prefer not to say |  |

*(ALL)

A2 Turning now to various areas of your life. How satisfied are you with…? Record number (Allowable range = 0 to 10)

*Please use a scale from 0-10, where 0 is completely dissatisfied and 10 is completely satisfied).*

*Please provide a response for each statement.*

|  | During the current coronavirus restrictions |
| --- | --- |
| your standard of living |  |
| your health |  |
| what you are currently achieving in life |  |
| your personal relationships |  |
| how safe you feel |  |
| feeling part of your community |  |
| your future security |  |

98. Not sure

99. Prefer not to say

*(ALL)

A4 Now a question about your wellbeing, during the **last month**, how often did you feel…

(STATEMENTS)

a) Nervous?

b) Hopeless?

c) Restless or fidgety?

d) So depressed that nothing could cheer you up?

e) That everything was an effort?

f) Worthless?

(RESPONSE FRAME)

1. All of the time

2. Most,

3. Some,

4. A little, or

5. None of the time

98. Not sure

99. Prefer not to say

**MODULE B: PHYSICAL ACTIVITY**

*(ALL)

B1 Now some questions about physical activity. Overall, do you feel you are doing more, less or about the same level of physical activity now – during the current coronavirus restrictions, compared to earlier in the year **before** any coronavirus restrictions began?

*Please select an option*

1. A lot more now

2. A little more now

3. About the same

4. A little less now

5. A lot less now

98. Not sure

99. Prefer not to say

*(B1=4 OR 5, DOING LESS PHYSICAL ACTIVITY)

B2 What is the main reason your physical activity level has been less during the current coronavirus restrictions?

*Please select all that apply*

1. Low motivation

2. Poor health or injury

3. Having less time

4. I have no-one to exercise with

5. Nowhere to exercise at home

6. More childcare responsibilities

7. No suitable park or path for physical activity outside

8. I’ve been concerned about catching coronavirus

9. I don’t feel safe being physically active outside

10. Having to wear a mask

11. One hour limit for outdoor physical activity

12. 8pm/9pm-5am curfew

13. 5km zone travel restriction

14. Can only exercise with one other person

15. Other (please specify)

98. Not sure *(EXCLUSIVE)

99. Prefer not to say *(EXCLUSIVE)

*(B1=1, 2 OR 3, DOING MORE OR SAME PHYSICAL ACTIVITY)

B3 What is the main reason your physical activity level has been more (or same) during the current coronavirus restrictions?

*Please select all that apply*

1. Having more time

2. I like catching up with others whilst exercising

3. I like my local area

4. I have more flexible work arrangements

5. Less childcare responsibilities

6. To get out of the house

7. I wanted to improve my health in general

8. I felt lonely

9. Other (please specify)

98. Not sure *(EXCLUSIVE)

99. Prefer not to say *(EXCLUSIVE)

*(ALL)

B4 In a usual week during the current coronavirus restrictions, on how many days do you do a total of 30 minutes or more of physical activity, which was enough to raise your breathing rate?

|  | During the current coronavirus restrictions |
| --- | --- |
| 0 |  |
| 1 |  |
| 2 |  |
| 3 |  |
| 4 |  |
| 5 |  |
| 6 |  |
| 7 |  |

98. Not sure

99. Prefer not to say

*(B4=1-7, DOES SOME KIND OF PHYSICAL ACTIVITY)

B5 Have you done any of the following activities during the current coronavirus restrictions?

|  | During the current coronavirus restrictions |
| --- | --- |
| Walking |  |
| Cycling |  |
| Running |  |
| Muscle strengthening exercises at home |  |
| Yoga/Pilates/stretching at home |  |
| Fitness/aerobics class at home |  |
| Online training sessions with local sports clubs |  |
| None of the above |  |
| 98. Not sure |  |
| 99. Prefer not to say |  |

**MODULE C: CONNECTING WITH OTHERS**

*(ALL)

C1 Please rate the degree to which you agree or disagree with the following statement:

I feel connected with others

|  | During the current coronavirus restrictions |
| --- | --- |
| Strongly disagree |  |
| Disagree |  |
| Mildly disagree |  |
| Mildly agree |  |
| Agree |  |
| Strongly agree |  |
| 98. Not sure |  |
| 99. Prefer not to say |  |

*(ALL)

C2 To what extent do you currently agree with the following statements…?

*Please provide a response for each statement.*

(STATEMENTS)

a) I am proud to be a member of my community

b) I feel I am part of the community

c) People in my neighbourhood share the same values

d) My neighbourhood is a good place to live

e) I trust my neighbours

f) People work together to get things done for this community

g) My neighbours are helping each other get through the current coronavirus restrictions

(RESPONSE FRAME)

1. Strongly agree

2. Agree

3. Neither agree nor disagree

4. Disagree

5. Strongly disagree

98. Not sure

99. Prefer not to say

*(ALL)

C4W Since the coronavirus restrictions started, how easy has it been to stay connected with family and friends outside your household?

1. Very easy

2. Easy

3. Neither easy nor hard

4. Hard

5. Very hard

98. Not sure

99. Prefer not to say

*(ALL)

C6 Are you involved with any community groups?

Please include groups such as sports clubs, book clubs, cultural groups, religious groups, fitness/exercise groups, and any related groups.

*Please select an option*

1. Yes

2. No

98. Not sure

99. Prefer not to say

*(C6=1, INVOLVED IN COMMUNITY GROUPS)

C7 How many community groups are you involved in?

*Please enter a response*

1. Record number of groups *(RECORD NUMBER BETWEEN 0 AND 50)

98. Not sure

99. Prefer not to say

*(C6=1, INVOLVED IN COMMUNITY GROUPS)

C8 Which of the following community groups or committees were you involved in earlier in the year before any coronavirus restrictions began, and which ones are you involved in now, during the current coronavirus restrictions?

*Please select all that apply for earlier in the year before any coronavirus restrictions began and during the current coronavirus restrictions.*

|  | a) Earlier in the year before any coronavirus restrictions began | b) During the current coronavirus restrictions |
| --- | --- | --- |
| 1. Sports club |  |  |
| 2. Community social benefit group (e.g. charity) |  |  |
| 3. Book club |  |  |
| 4. School/kindergarten/crèche volunteer group |  |  |
| 5. Parents of young children group/mothers group |  |  |
| 6. Education/study groups |  |  |
| 7. Environmental group |  |  |
| 8. Informal exercise group |  |  |
| 9. Formal fitness class/group |  |  |
| 10. Online social/gaming group |  |  |
| 11. Arts group |  |  |
| 12. Music group |  |  |
| 13. Dance group |  |  |
| 14. Religious group |  |  |
| 15. Cultural/ethnic group |  |  |
| 16. Political group |  |  |
| 17. Hobby group |  |  |
| 1. Other (Please specify) |  |  |
| 1. None of the above |  |  |
| 1. Not sure |  |  |
| 1. Prefer not to say |  |  |

*(COMMUNITY GROUPS SELECTED IN BOTH C8a AND C8b, INVOLVED BOTH BEFORE AND NOW)

C9 How has your level of involvement in the following community groups changed during the current coronavirus restrictions, compared to earlier in the year **before** any coronavirus restrictions began?

(STATEMENTS) (ONLY SHOW IF SELECTED AT C8a AND C8b)

a) Sports club

b) Community social benefit group (e.g. charity)

c) Book club

d) School/kindergarten/crèche volunteer group

e) Parents of young children group/mothers group

f) Education/study groups

g) Environmental group

h) Informal exercise group

i) Formal fitness class/group

j) Online social/gaming group

k) Arts group

l) Music group

m) Dance group

n) Religious group

o) Cultural/ethnic group

p) Political group

q) Hobby group

r) Other

(RESPONSE FRAME)

1. A lot more now

2. A little more now

3. About the same

4. A little less now

5. A lot less now

98. Not sure

99. Prefer not to say

*(ALL)

C10 Do you plan to be involved in any of the following once the coronavirus restrictions are over?

(STATEMENTS)

a) Sports club

b) Community social benefit group (e.g. charity)

c) Book club

d) School/kindergarten/crèche volunteer group

e) Parents of young children group/mothers group

f) Education/study groups

g) Environmental group

h) Informal exercise group

i) Formal fitness class/group

j) Online social/gaming group

k) Arts group

l) Music group

m) Dance group

n) Religious group

o) Cultural/ethnic group

p) Political group

q) Hobby group

r) Other

(RESPONSE FRAME)

1. Yes

2. No

98. Not sure

99. Prefer not to say

**MODULE D: HEALTHY EATING**

*(ALL)

D1 During the current coronavirus restrictions, how many serves of vegetables are you usually eating each day?

*A ‘serve’ is ½ cup of cooked vegetables or 1 cup of salad vegetables.*

*‘Vegetables’ includes potatoes, hot potato chips, but excludes potato crisps and vegetable juice.*

*Please enter a response.*

1. Record number of serves *(RECORD NUMBER BETWEEN 0 AND 50)

98. Not sure

99. Prefer not to say

*(ALL)

D2 Overall, do you feel you are eating more, less or about the same amount of vegetables now – during the current coronavirus restrictions, compared to earlier in the year **before** any coronavirus restrictions began?

*Please select an option*

1. A lot more now

2. A little more now

3. About the same

4. A little less now

5. A lot less now

98. Not sure

99. Prefer not to say

*(D2=CODES 1 OR 2, EATING MORE VEGETABLES DURING COVID)

D2a What is the main reason you’ve eaten more vegetables during the current coronavirus restrictions?

*Please select all that apply*

1. I’m cooking more

2. I have more time

3. I’ve learnt new ways to prepare or cook them

4. I want to look after my health more than before

5. Other (please specify)

98. Not sure *(EXCLUSIVE)

99. Prefer not to say *(EXCLUSIVE)

*(D2=CODES 4 OR 5, EATING LESS VEGETABLES DURING COVID)

D2b What is the main reason you’ve eaten less vegetables during the current coronavirus restrictions?

*Please select all that apply*

1. They’re too expensive

2. I don’t like them

3. It’s easier to prepare other food

4. I can’t get the vegetables I usually buy

5. Other (please specify)

98. Not sure *(EXCLUSIVE)

99. Prefer not to say *(EXCLUSIVE)

*(ALL)

N1 During the current coronavirus restrictions, how many glasses of soft drink, cordial, flavoured mineral water, energy drink or sports drink are you consuming every day (excluding diet variety)?

1. None

2. Less than 1 per day

3. 1-2 per day

4. 3-4 per day

5. 5+ per day

98. Not sure

99. Prefer not to say

*(ALL)

N2 Overall, do you feel you are drinking more, less or about the same amount of soft drink, cordial, flavoured mineral water, energy drink or sports drink now – during the current coronavirus restrictions, compared to earlier in the year **before** any coronavirus restrictions began?

*Please select an option*

1. A lot more now

2. A little more now

3. About the same

4. A little less now

5. A lot less now

98. Not sure

99. Prefer not to say

*(N2=CODES 4 OR 5, DRINKING LESS SSBs)

N2a What is the main reason you’ve been drinking less sugary drinks during the current coronavirus restrictions?

*Please select all that apply*

1. They’re too expensive

2. They’re not good for my health

3. I don’t keep them at home

4. Other (please specify)

98. Not sure *(EXCLUSIVE)

99. Prefer not to say *(EXCLUSIVE)

*(N2=CODES 1 TO 3, DRINKING MORE SSBs)

N2b What is the main reason you’ve had more (or same) sugary drinks during the current coronavirus restrictions?

*Please select all that apply*

1. It’s a treat

2. I enjoy it

3. I drink them when I’m bored

4. They’re easy to buy

5. I’ve been buying it for others in my household

6. I’ve been ordering more takeaway and getting soft drinks with it

7. It’s always available at home

8. They were on sale/discounted

9. Other (please specify)

98. Not sure *(EXCLUSIVE)

99. Prefer not to say *(EXCLUSIVE)

*(ALL)

N3 During the current coronavirus restrictions, how often are you having meals or snacks such as burgers, pizza, chicken or chips from places like McDonalds, Hungry Jacks, Pizza Hut, KFC, Red Rooster, or local take-away places?

*Please do not include sushi, take-away Asian foods, salads, sandwiches or rolls*

1. Most days (6-7 times per week)

2. 3-5 times per week

3. 1-2 times per week

4. 2-3 times per month

5. Once per month

6. Less than once per month

7. Never

98. Not sure

99. Prefer not to say

*(ALL)

N4 Overall, do you feel you are having more, less or about the same number of meals or snacks such as burgers, pizza, chicken or chips from places like McDonalds, Hungry Jacks, Pizza Hut, KFC, Red Rooster, or local take-away places now – during the current coronavirus restrictions, compared to earlier in the year **before** any coronavirus restrictions began?

*Please do not include sushi, take-away Asian foods, salads, sandwiches or rolls*

*Please select an option*

1. A lot more now

2. A little more now

3. About the same

4. A little less now

5. A lot less now

98. Not sure

99. Prefer not to say

*(N4=CODES 4 OR 5, HAD LESS TAKEAWAY)

N4a What is the main reason you’ve had less take-away food during the current coronavirus restrictions?

*Please select all that apply*

1. They’re too expensive

2. They’re not good for my health

3. It’s too hard to buy

4. I have more time to cook meals

5. I’m concerned I’ll get coronavirus

96. Other (Please specify)

98. Not sure *(EXCLUSIVE)

99. Prefer not to say *(EXCLUSIVE)

*(N4=CODES 1 TO 3, HAD MORE TAKEAWAY)

N4b What is the main reason you’ve had more (or same) take away food during the current coronavirus restrictions?

*Please select all that apply*

1. It’s a treat

2. Something to break up the week

3. I don’t have enough time to cook

4. It’s easy to buy

5. It’s easier than cooking

6. They were on sale/discounted

7. Other (Please specify)

98. Not sure *(EXCLUSIVE)

99. Prefer not to say *(EXCLUSIVE)

*(ALL)

D3 Have you or anyone in your household **started** doing the following during the current coronavirus restrictions?

*Please provide a response for each statement.*

(STATEMENTS)

a) Planted vegetable seeds or seedlings or grown food

b) Purchased food from a farmers’ market, vegetable box scheme or local farm

c) Ordered a takeaway from an online delivery service (e.g. Deliveroo, Uber Eats etc.)

d) Ordered food directly from a local restaurant or cafe

e) Planned meals for the week

f) Kept more food and other essentials at home

g) Shopped locally, for example started going to local grocer, fruit and vegetable supply, butcher

(RESPONSE FRAME)

1. Yes

2. No

98. Not sure

99. Prefer not to say

*(IF YES TO ANY STATEMENT AT D3)

*(DISPLAY ONLY CODES ANSWERED YES IN D3)

D3a Do you plan to continue with any of the following after the current coronavirus restrictions are over?

*Please provide a response for each statement.*

(STATEMENTS)

*(DISPLAY ONLY CODES ANSWERED YES IN D3)

a) Plant vegetable seeds or seedlings or other grown food

b) Purchase food from a farmers’ market, vegetable box scheme or local farm

c) Order a takeaway from an online delivery service (e.g. Deliveroo, Uber Eats etc.)

d) Order food directly from a local restaurant or cafe

e) Plan meals for the week

f) Keep more food and other essentials at home

g) Shop locally, for example started going to local grocer, fruit and vegetable supply, butcher

(RESPONSE FRAME)

1. Yes

2. No

98. Not sure

99. Prefer not to say

*(ALL)

D4 On average, during the current COVID restrictions, how many times do you and your household cook dinner each week?

*Please enter a response*

1. Record number of meals *(RECORD NUMBER BETWEEN 0 AND 7)

98. Not sure

99. Prefer not to say

*(ALL)

D7a During the current coronavirus restrictions, did you have to rely on a restricted range of low-cost unhealthy food because you were running out of money to buy food?

1. No, not at all

2. Not often

3. Sometimes, or

4. Yes, definitely

98. Not sure

99. Prefer not to say

**MODULE E: ALCOHOL**

*(ALL)

E1 During the current coronavirus restrictions, how often have you had an alcoholic drink of any kind?

*Please select an option.*

1. Every day

2. 5 to 6 days a week

3. 3 to 4 days a week

4. 1 to 2 days a week

5. 2 to 3 days a month

6. About 1 day a month

7. Less often

8. I never drink alcohol

98. Not sure

99. Prefer not to say

*(DRINKS ALCOHOL, E1=1-7, 98, 99)

E2 Would you say this is more, less, or about the same now – during the current coronavirus restrictions, compared to earlier in the year **before** any coronavirus restrictions began?

*Please select an option*

1. A lot more now

2. A little more now

3. About the same

4. A little less now

5. A lot less now

98. Not sure

99. Prefer not to say

*(E2=1 OR 2, DRINKING ALCOHOL ON MORE DAYS)

E5 What is the main reason you’ve drank alcohol on *more days* during the current coronavirus restrictions?

*Please select ALL that apply*

1. I had more time

2. I was bored

3. I was anxious or stressed

4. I felt lonely

5. I had more income

6. I had less income

7. I didn’t need to stay below .05 for driving

8. The person/people I live with are drinking alcohol

9. Socialising online often involves alcohol

10. Other (please specify)

98. Not sure *(EXCLUSIVE)

99. Prefer not to say *(EXCLUSIVE)

*(E2=4 OR 5, DRINKING ALCOHOL ON LESS DAYS)

E6 What is the main reason you’ve drank alcohol on *less days* during the current coronavirus restrictions?

*Please select ALL that apply*

1. I had fewer opportunities to drink at home

2. I wanted to improve my health in general

3. The places where I usually drink are closed e.g. bars, clubs, restaurants

4. I was specifically concerned that drinking alcohol could increase the risk or severity of coronavirus

5. I can’t socialise with the people I usually drink with

6. I had more income

7. I had less income

8. Other (please specify)

98. Not sure *(EXCLUSIVE)

99. Prefer not to say *(EXCLUSIVE)

*(DRINKS ALCOHOL, E1=1-7, 98, 99)

E3 Still thinking about during the current coronavirus restrictions… On a day that you have an alcoholic drink, how many standard drinks do you usually have?

*A standard drink is equal to 1 pot of full-strength beer, 1 small glass of wine or 1 pub-sized nip of spirits.*

*Please select an option.*

1. 20 or more standard drinks

2. 16 – 19 standard drinks

3. 13 – 15 standard drinks

4. 11 – 12 standard drinks

5. 9 – 10 standard drinks

6. 7 – 8 standard drinks

7. 5 – 6 standard drinks

8. 3 – 4 standard drinks

9. 2 standard drinks

10. 1 standard drink

11. Half a standard drink

98. Not sure

99. Prefer not to say

*(DRINKS ALCOHOL, E1=1-7, 98, 99)

E4 Would you say this is more, less, or about the same now – during the current coronavirus restrictions, compared to earlier in the year **before** any coronavirus restrictions began?

*Please select an option*

1. A lot more now

2. A little more now

3. About the same

4. A little less now

5. A lot less now

98. Not sure

99. Prefer not to say

**MODULE F: SMOKING**

*(ALL)

F1 Now I’d like to ask you some questions about smoking. Do you now smoke cigarettes, cigars, pipes or any other tobacco products?

1. Daily

2. At least weekly (not daily)

3. Less often than weekly, or

4. Not at all

98. Not sure

99. Prefer not to say

*(CURRENT SMOKER (F1=1-3))

F2 During the current coronavirus restrictions, did you do any of the following?

1. Smoked *more* than usual 🡪 Go to QF3

2. Smoked *less* than usual 🡪 Go to F4

3. Attempted to quit Go to F5

4. Quit smoking 🡪 Go to QF6

5. Did not change my smoking behaviour 🡪 Go to G1

98. Not sure

99. Prefer not to say

*(F2=1, SMOKING MORE)

F3 What is the main reason you smoked *more* than usual during the current coronavirus restrictions?

*Please select ALL that apply*

1. I had more time

2. I was bored

3. I was anxious or stressed

4. I had more disposable income

5. I felt lonely

6. Other (please specify)

98. Not sure *(EXCLUSIVE)

99. Prefer not to say *(EXCLUSIVE)

*(F2=2, SMOKING LESS)

F4 What is the main reason you smoked *less* than usual during the current coronavirus restrictions?

*Please select ALL that apply*

1. I had fewer opportunities to smoke at home

2. I wanted to improve my health in general

3. I was specifically concerned that smoking could increase the risk or severity of coronavirus

4. My income was reduced

5. Other (please specify)

98. Not sure *(EXCLUSIVE)

99. Prefer not to say *(EXCLUSIVE)

*(F2=3, TRIED TO QUIT)

F5 What is the main reason you attempted to quit during the current coronavirus restrictions?

*Please select ALL that apply*

1. I had fewer opportunities to smoke at home

2. I tried to quit smoking to improve my health in general

3. I was specifically concerned that smoking could increase the risk or severity of coronavirus

4. I tried to quit smoking to save money, as my income was reduced

5. I tried to quit smoking because the cost of cigarettes/tobacco went up

6. Other (please specify)

98. Not sure *(EXCLUSIVE)

99. Prefer not to say *(EXCLUSIVE)

*(F2=4, QUIT)

F6 What is the main reason you’ve quit during the current coronavirus restrictions?

*Please select ALL that apply*

1. I had fewer opportunities to smoke at home

2. I quit smoking to improve my health in general

3. I was specifically concerned that smoking could increase the risk or severity of coronavirus

4. I quit smoking to save money, as my income was reduced

5. I quit smoking because the cost of cigarettes/tobacco went up

6. Other (please specify)

98. Not sure *(EXCLUSIVE)

99. Prefer not to say *(EXCLUSIVE)

**MODULE G: WORKING AND HOME LIFE DURING COVID**

*(ALL)

G1 Now we are going to ask some questions about your home life. Which of these best describes your household…?

1. Person living alone

2. Couple living alone

3. Couple with child / children

4 One parent family with child / children, co-parenting with other parent living elsewhere

5 One parent family with child / children

6. Adults sharing house /apartment / flat

96. Something else (please specify)

98. Not sure

99. Prefer not to say

*(ALL)

G1a Which of these best describes your current main activity? Are you…? / And how about your partner?

|  | *(ALL) | *(G1=CODES 2 OR 3, HAS PARTNER AT HOME) |
| --- | --- | --- |
|  | 1. Which of these best describes your main activity since coronavirus restrictions started? Are you…? | 1. And how about your partner? |
| 1. Self employed |  |  |
| 2. Employed for wages, salary or payment in kind |  |  |
| 3. Unemployed |  |  |
| 4. Engaged in home duties |  |  |
| 5. A student |  |  |
| 6. Retired |  |  |
| 7. Unable to work |  |  |
| 96. Something else (please specify) |  |  |
| 98. Not sure |  |  |
| 99. Prefer not to say |  |  |

*(NEW)

G2 Which of these best describes your main activity in February 2020? Were you…?

1. Self employed

2. Employed for wages, salary or payment in kind

3. Unemployed

4. Engaged in home duties

5. A student

6. Retired

7. Unable to work

96. Something else (please specify)

98. Not sure

99. Prefer not to say

*(G2=1-2, HAD JOB IN FEBRUARY 2020)

G3 And in February 2020, how many hours did you do in your job?

*If you had more than one job, please enter the number of hours for ALL your jobs.*

1. Enter number of hours (ALLOWABLE RANGE: 1-100)

98. Not sure

99. Prefer not to say

*(G2=1-2, HAD JOB IN FEBRUARY 2020)

G4 What industry did you work in for your main job in February 2020?

*If you had more than one job, please enter the usual place of work for your MAIN job.*

1. Agriculture, forestry and fishing

2. Mining

3. Manufacturing

4. Electricity, gas, water and waste services

5. Construction

6. Wholesale trade

7. Retail trade

8. Accommodation and food services (e.g., hotels, cafes, restaurants, pubs, takeaway)

9. Transport, postal and warehousing

10. Information media and telecommunications

11. Financial and insurance services

12. Rental, hiring and real estate services

13. Professional, scientific and technical services

14. Administrative and support services

15. Public administration and safety

16. Education and training

17. Health care and social assistance

18. Arts services

19. Sports and recreation services

20. Something else (please specify)

98. Not sure

99. Prefer not to say

*(G2=1-2, HAD JOB IN FEBRUARY 2020)

G5 And in February 2020, where was your usual place of work?

*If you had more than one job, please enter the usual place of work for your MAIN job.*

1. Worked mainly from home with standard hours

2. Worked mainly from home with flexible start and finish times

3. Worked mainly from another location e.g. office with standard hours

4. Worked mainly from another location e.g. office with flexible start and finish times

98. Not sure

99. Prefer not to say

*(ALL)

G6 Thinking now about since the coronavirus restrictions started, have **you** experienced any of the following?

(STATEMENTS)

a) Had your hours of work reduced

b) Your hourly rate of pay / salary been reduced not related to the number of hours you work

c) Not received a bonus that you were entitled to

d) Lost your job

e) Required to take paid leave

f) Required to take unpaid leave

g) The company you worked for ceased operating / had to close my business

(RESPONSE FRAME)

1. Yes

2. No

98. Not sure *(EXCLUSIVE)

99. Prefer not to say *(EXCLUSIVE)

*(G1a=1 OR 2, CURRENTLY EMPLOYED)

G7 During the current COVID -19 restrictions, where is your usual place of work?

|  | 1. During the current coronavirus restrictions, where is your usual place of work?   *If you had more than one job, please enter the usual place of work for your MAIN job* |
| --- | --- |
| 1. Worked mainly from home with standard hours |  |
| 2. Worked mainly from home with flexible start and finish times |  |
| 3. Worked mainly from another location e.g. office with standard hours |  |
| 4. Worked mainly from another location e.g. office with flexible start and finish times |  |
| 98. Not sure |  |
| 99. Prefer not to say |  |

*(ALL)

G7a Since coronavirus restrictions started which of the following apply to you, if any?

*Please select ALL that apply*

1. Received, or have been notified that you will receive JobKeeper

2. Received, or have been notified that you will receive JobSeeker

97. None of these *(EXCLUSIVE)

98. Not sure *(EXCLUSIVE)

99. Prefer not to say *(EXCLUSIVE)

*(G1=3, 4 OR 5, HOUSEHOLD STRUCTURE HAS CHILDREN)

G8 Thinking about your household, how many children aged under 18, if any, live in your household (at least 50% of the time)?

1. Number of children given (please specify) *(ALLOWABLE RANGE 1-20)

2. None

98. Not sure *(EXCLUSIVE)

99. Prefer not to say *(EXCLUSIVE)

*(G8=1, HAS DEPENDENT CHILDREN IN HOUSEHOLD)

*(IF G8=1 IS 1, ONE DEPENDENT CHILD IN HOUSEHOLD, SHOW ‘child’.

IF G8=1 IS <1, MORE THAN ONE DEPENDENT CHILDREN IN HOUSEHOLD, SHOW ‘child with the most recent birthday’.)

G8a Thinking about your <child/child with the most recent birthday>, how old are they?

1. Age of <child/child with most recent birthday> (please specify) *(ALLOWABLE RANGE 0-18)

99. Prefer not to say *(EXCLUSIVE)

*(G8=1, has dependent children in household)

G9 Which of the following applied to you during most of the current coronavirus restrictions?

*Please select ALL that apply*

1. I have kept my child/children in childcare or kindergarten

2. I have started my child/children in childcare or kindergarten

3. I have discontinued my child/children going to childcare or kindergarten

4. I was unable to send my child/children to childcare or kindergarten because the centre(s) was shut down as a result of coronavirus restrictions

5. I have child/children at school

6. I have child/children doing school at home

97. None of these *(EXCLUSIVE)

98. Not sure *(EXCLUSIVE)

99. Prefer not to say *(EXCLUSIVE)

*(CHILDREN DISCONTINUED CHILDCARE, G9=3 OR 4 AND G1=3 OR 4, ANOTHER PARENT INVOLVED)

G10 Who would you say is spending, or has spent, the most time looking after your preschool child(ren) during the current coronavirus restrictions?

1. I am

2. My partner or other parent

3. Shared equally between my partner / the other parent and myself

4. Someone else in the household (please specify)

98. Not sure *(EXCLUSIVE)

99. Prefer not to say *(EXCLUSIVE)

*(CHILDREN DOING HOME SCHOOLING, G9=6 AND G1=3 OR 4, ANOTHER PARENT INVOLVED)

G11 Who would you say is spending, or has spent, the most time helping your child(ren) with school at home during the current coronavirus restrictions?

1. I am

2. My partner or other parent

3. Shared equally between my partner / the other parent and myself

4. Someone else in the household (please specify)

98. Not sure *(EXCLUSIVE)

99. Prefer not to say *(EXCLUSIVE)

*(G8=1, HAS DEPENDENT CHILDREN IN HOUSEHOLD)

*(IF G8=1 IS 1, ONE DEPENDENT CHILD IN HOUSEHOLD, SHOW ‘child’.

IF G8=1 IS <1, MORE THAN ONE DEPENDENT CHILDREN IN HOUSEHOLD, SHOW ‘child aged [INSERT AGE FROM G8a]’.

IF G8a=99, PREFER NOT TO SAY AGE OF CHILD, SHOW ‘child with the most recent birthday’)

G17 Thinking about your <child/child aged [INSERT AGE FROM G8a]/child with the most recent birthday >, during the current coronavirus restrictions, how many glasses of soft drink, cordial, flavoured mineral water, energy drink or sports drink does your child consume every day (exclude diet variety)?

*(IF G8=2 OR MORE CHILDREN UNDER 18) *Please think about your child with the most recent birthday.*

1. None

2. Less than 1 per day

3. 1-2 per day

4. 3-4 per day

5. 5+ per day

98.     Not sure

99.     Prefer not to say

*(G8=1, HAS DEPENDENT CHILDREN IN HOUSEHOLD)

G17a And would you say this is more, less or about the same as earlier in the year **before** any coronavirus restrictions began?

*Please select an option.*

1. A lot more now

2. A little more now

3. About the same

4. A little less now

5. A lot less now

98.     Not sure

99.     Prefer not to say

*(G8=1, HAS DEPENDENT CHILDREN IN HOUSEHOLD)

G18 During the current coronavirus restrictions, how often does your child have meals or snacks such as burgers, pizza, chicken or chips from places like McDonalds, Hungry Jacks, Pizza Hut, KFC, Red Rooster, or local take-away places?

*Please do not include sushi, take-away Asian foods, salads, sandwiches or rolls*

*(IF G8=2 OR MORE CHILDREN UNDER 18) *Please think about your child with the most recent birthday.*

1. Most days (6-7 times per week)

2. 3-5 times per week

3. 1-2 times per week

4. 2-3 times per month

5. Once per month

6. Less than once per month

7. Never

98. Not sure

99. Prefer not to say

*(G8=1, HAS DEPENDENT CHILDREN IN HOUSEHOLD)

G18a And would you say this is more, less or about the same as earlier in the year **before** any coronavirus restrictions began?

*Please select an option*

1. A lot more now

2. A little more now

3. About the same

4. A little less now

5. A lot less now

98.     Not sure

99.     Prefer not to say

*(G8=1, HAS DEPENDENT CHILDREN IN HOUSEHOLD)

G20 During the current coronavirus restrictions, how many times a day does your child eat snack foods (e.g. chips, shapes, crackers, sweet biscuits, muesli bars or cakes)?

*Please enter a response*

1. Record number of times *(RECORD NUMBER BETWEEN 0 AND 50)

98.     Not sure

99.     Prefer not to say

*(G8=1, HAS DEPENDENT CHILDREN IN HOUSEHOLD)

G21 And would you say this is more, less or about the same as earlier in the year **before** any coronavirus restrictions began?

*Please select an option*

1. A lot more now

2. A little more now

3. About the same

4. A little less now

5. A lot less now

98.     Not sure

99.     Prefer not to say

*(G8=1, HAS DEPENDENT CHILDREN IN HOUSEHOLD)

G19 During the current coronavirus restrictions, in a usual week, on how many days does your child do a total of one hour or more of physical activity, which was enough to raise their breathing rate?

|  | During the current coronavirus restrictions |
| --- | --- |
| 0 |  |
| 1 |  |
| 2 |  |
| 3 |  |
| 4 |  |
| 5 |  |
| 6 |  |
| 7 |  |
| 98. Not sure |  |
| 99. Prefer not to say |  |

*(G8=1, HAS DEPENDENT CHILDREN IN HOUSEHOLD)

G19a And would you say this is more, less or about the same as earlier in the year **before** any coronavirus restrictions began?

*Please select an option*

1. A lot more now

2. A little more now

3. About the same

4. A little less now

5. A lot less now

98.     Not sure

99.     Prefer not to say

*(ALL)

G12 Since coronavirus restrictions began, did any of the following happen because of a shortage of money?

|  | **During the current coronavirus restrictions** |
| --- | --- |
| a. Could not pay electricity, gas or telephone bills on time |  |
| b. Could not pay the rent or mortgage on time |  |
| c. Pawned or sold something (Definition of ‘pawned’ – when an individual receives money for their personal property (e.g. Cash Converters)) |  |
| d. Went without meals |  |
| e. Asked for financial help from friends or family |  |
| f. Asked for help from welfare/community organisations |  |
| g. Attended a food relief agency, food bank or food pantry (or similar) to access food relief |  |
| h. Worried about having enough money to buy food |  |
| i. Skipped a meal in order to feed your household |  |
| j. Ran out of food and could not afford to buy more |  |
| k. Applied for early access to my superannuation |  |

(RESPONSE FRAME)

1. Yes

2. No

98. Not sure

99. Prefer not to say

*(ALL)

G13 Thinking about how you feel right now, on a scale of 1 to 5, where 1 is very concerned and 5 is not at all concerned, would you say...?

(STATEMENTS)

a) I feel concerned about my future employment/job prospects

b) I feel concerned about the stability of my housing

c) I feel concerned about my loss of connection to others outside my household

(RESPONSE FRAME)

1. 1 – Very concerned

2. 2

3. 3

4. 4

5. 5 – Not at all concerned

98. Not sure

99. Prefer not to say

*(ALL)

G14 Please identify if you or those you know have been diagnosed with coronavirus?

1. Self

2. Close family member

3. Family member

4. Close friend

5. Friend

6. Household member

7. Work colleague

8. Recent acquaintance

9. I don’t know anyone who has been diagnosed with coronavirus

98. Not sure

99. Prefer not to say

*(ALL)

G15 Some people have found that some of the changes made during the coronavirus pandemic have been positive.

Thinking about your **work life, social life, home life and your wellbeing**, are there any aspects from the coronavirus period that you would like to maintain after restrictions are over?

*Please write in your response to each of the following:*

| **Life area** | **Yes, please tell us what changes you would like to keep** | **No** | **Not sure** | **Prefer not to say** |
| --- | --- | --- | --- | --- |
| Work life (e.g. work from home, change my job, ask for flexible hours) |  |  |  |  |
| Social life (e.g. walking with friends, using zoom or facetime to talk to friends,  see more of my neighbours) |  |  |  |  |
| Home life (e.g. spend more time with my children, do more with my household/family, keep doing gardening) |  |  |  |  |
| Personal wellbeing (e.g. keep exercising, look after my health, meditate) |  |  |  |  |

*(ALL)

G22 Could you describe any other impacts, positive or negative, that the outbreak of coronavirus has had on your life?

**Positive impacts**

(INSERT OPEN-END TEXT BOX)

**Negative impacts**

(INSERT OPEN-END TEXT BOX)

**MODULE S: SOCIO-DEMOGRAPHICS AND OTHER COVARIATES**

*(NEW)

H1 Where were you located during the 2019/2020 summer bushfires?

*Please select one option*

1. Community member in bushfire affected area

2. Holidaying in or travelling through bushfire affected area

3. Not located in a bushfire affected area

98. Not sure *(EXCLUSIVE)

99. Prefer not to say *(EXCLUSIVE)

*(NEW)

H2 To what degree would you say you were affected by the 2019/2020 summer bushfires?

1. Not affected at all

2. Slightly affected

3. Affected a fair amount

4. Severely affected

98. Not sure *(EXCLUSIVE)

99. Prefer not to say *(EXCLUSIVE)

*(NEW)

S1W Now I have some questions to help us analyse the results. Just to confirm, what gender do you identify as?

1. Male

2. Female

3. Non-binary

96. Other

98. Not sure *(EXCLUSIVE)

99. Prefer not to say *(EXCLUSIVE)

*(ALL)

S2W How old were you last birthday?

1. Age given *(RECORD AGE IN YEARS – ALLOWABLE RANGE 18 TO 99)

99. Prefer not to say *(EXCLUSIVE)

*(G2=99, REFUSED AGE)

S3W Which of the following broad age groups are you in?

1. 18 – 24 years

2. 25 – 34 years

3. 35 – 44 years

4. 45 – 54 years

5. 55 – 64 years

6. 65 – 74 years

7. 75+ years

99. Prefer not to say *(EXCLUSIVE)

*(ALL)

S4W What is your postcode?

1. Record postcode

98. Not sure *(EXCLUSIVE)

99. Prefer not to say *(EXCLUSIVE)

*(S4=98 OR 99, REFUSED POSTCODE)

S5 Would you be happy to provide your locality or suburb?

1. Record locality

98. Not sure *(EXCLUSIVE)

99. Prefer not to say *(EXCLUSIVE)

*(ALL)

S6W Which of the following best describes your housing situation?

1. Own outright

2. Own with a mortgage

4. Renting

5. Occupying rent free

3. Purchasing under a shared equity scheme (A shared equity scheme is a way to share the cost of buying a home with an equity partner, such as a private investor, not-for profit organisation or government housing authority.)

6. Occupying under a life tenure scheme (A life tenure scheme is a contract to live in the dwelling for the term of your life without the full rights of ownership. This is a common arrangement in retirement villages.)

7. Some other arrangement (please specify)

98. Not sure *(EXCLUSIVE)

99. Prefer not to say *(EXCLUSIVE)

*(ALL)

S7W Which of the following best describes your current relationship status? Are you…?

1. Married

2. Living with a partner

3. Widowed

4. Divorced

5. Separated

6. Never married

98. Not sure *(EXCLUSIVE)

99. Prefer not to say *(EXCLUSIVE)

*(NEW)

S8W Are you of Aboriginal or Torres Strait Islander origin?

1. No, not Aboriginal or Torres Strait Islander

2. Yes, Aboriginal

3. Yes, Torres Strait Islander

4. Yes, Aboriginal and Torres Strait Islander

98. Not sure *(EXCLUSIVE)

99. Prefer not to say *(EXCLUSIVE)

*(NEW)

S9W In which country were you born?

1. Australia (includes External Territories)

2. United Kingdom (incl. England, Scotland, Wales, Northern Ireland)

3. New Zealand

4. Italy

5. Greece

6. China

7. Vietnam

8. Lebanon

9. India

10. Philippines

96. Other (please specify)

98. Not sure *(EXCLUSIVE)

99. Prefer not to say *(EXCLUSIVE)

*(NEW)

S10W Do you speak a language other than English at home?

1. Yes

2. No

98. Not sure (EXCLUSIVE)

99. Prefer not to say (EXCLUSIVE)

*(NEW)

S11W What is the highest year of schooling you have completed?

1. Year 12 or equivalent

2. Year 11 or equivalent

3. Year 10 or equivalent

4. Years 7-9 or equivalent

5. Completed primary school but did not go to high school

6. Some primary school only

7. Did not go to school

98. Not sure (EXCLUSIVE)

99. Prefer not to say (EXCLUSIVE)

*(NEW)

S12W What is the highest post-school educational qualification that you have obtained?

*Apprenticeship can be coded to Cert III or IV. Traineeship can usually be coded to Cert I or II.*

1. No post school educational qualification

2. Certificate I or Certificate II

3. Certificate III or Certificate IV

4. Associate Diploma

5. Undergraduate Diploma

6. Bachelor Degree

7. Master’s Degree, Postgraduate Degree or Postgraduate Diploma

8. Doctorate

96. Other (please specify)

98. Not sure (EXCLUSIVE)

99. Prefer not to say (EXCLUSIVE)

*(ALL)

S13aW Which of the following ranges best describes your <personal / household> approximate income, from all sources, before tax is taken out, up to February 2020? Please include wages and salaries, government pensions, benefits and allowances, and income from interest, dividends or other sources.

*(PROGRAMMER NOTE: IF G1=1 or 6, USE PERSONAL, ELSE USE HOUSEHOLD’S)

1. Less than $10,000

2. $10,000 – less than $20,000

3. $20,000 – less than $30,000

4. $30,000 – less than $40,000

5. $40,000 – less than $50,000

6. $50,000 – less than $60,000

7. $60,000 – less than $80,000

8. $80,000 – less than $100,000

9. $100,000 – less than $125,000

10. $125,000 – less than $150,000

11. $150,000 – to less than $200,000

12. $200,000 or more

98. Not sure (EXCLUSIVE)

99. Prefer not to say (EXCLUSIVE)

*(ALL)

S13b Is your income more, less or the same now – during the current coronavirus restrictions, compared to earlier in the year **before** any coronavirus restrictions began?

*Please select an option.*

1. A lot more now

2. A little more now

3. About the same

4. A little less now

5. A lot less now

98. Not sure (EXCLUSIVE)

99. Prefer not to say (EXCLUSIVE)

*(ALL)

P_DISABILITYW Do you currently have a disability, health condition or injury that has lasted, or is likely to last, 6 months or more which restricts your everyday activities?

1. Yes

2. No

98. Not sure

99. Prefer not to say

*(ALL)

S21W Other than a Medicare card, are you the holder of a health care card or a pensioner concession card?

*Health care cards are issued by Centrelink and are different to Medicare cards.*

1. Yes

2. No

98. Not sure

99. Prefer not to say

*(ALL)

S22 In order to analyse the results of this survey at a local level, we’d like to make a note of the nearest cross street intersection to your house. This information will only be used so we can join your answers with others in your neighbourhood. It will not be used to identify you. Are you able to give me the nearest cross street intersection?

1. Suburb

2. Postcode

3. Cross streets given (specify Street One and Street Two separately)

98. Not sure *(EXCLUSIVE)

99. Prefer not to say *(EXCLUSIVE)

*(ALL)

R1 Would you be happy to be recontacted to take part in a similar survey in the future?

1. Yes

2. No

99. Prefer not to say
