# Supplementary material for: A repeated cross-sectional and longitudinal study of mental health and wellbeing during COVID-19 lockdowns in Victoria, Australia
Source: BMC Public Health. 2022 Dec 27;22:2434. doi: 10.1186/s12889-022-14836-9 (PMC9793381; doi:10.1186/s12889-022-14836-9)
Supplement: Supplementary file 2 — Additional file 2: Table S1. Cohort characteristics at the first lockdown & the second lockdowns. Table S2. Baseline characteristics of the cohort with and without missing at the second lockdown. Table S3. Results from univariable logistic regression modelling with psychological distress and life satisfaction asoutcome at the first lockdown**. Table S4. Results from univariable logistic regression modelling with psychological distress and life satisfaction as outcome at the second lockdown**. Table S5. Longitudinal associations of change in psychological distress and life satisfaction between the first and the second lockdowns**. [file 12889_2022_14836_MOESM2_ESM.docx]

**Supplementary File 2**

Table S1: Cohort characteristics at the first lockdown & the second lockdowns

|  | First lockdown | Second lockdown |
| --- | --- | --- |
|  | N=2,000  N (%) or  mean (SD)^#^ | N=2,000  N (%) or  mean (SD)^#^ |
| Social Solidarity | 21.33 (4.42) | 21.05 (4.55) |
| ^*^Feeling connected with others |  |  |
| Agree | 785 (39.3%) | 666 (33.3%) |
| Mildly | 661 (33.1%) | 701 (35.0%) |
| Disagree | 435 (21.8%) | 546 (27.3%) |
| Missing | 119 (5.9%) | 87 (4.3%) |
| ^*^Staying connected with family and friends |  |  |
| Easy | 749 (37.5%) | 569 (28.4%) |
| Neither | 609 (30.4%) | 549 (27.5%) |
| Hard | 574 (28.7%) | 844 (42.2%) |
| Missing | 68 (3.4%) | 38 (1.9%) |
| ^*^Life Satisfaction |  |  |
| Low/Medium (Rated 0 to 6) | 937 (46.9%) | 1,062 (53.1%) |
| High (Rated 7 or 10) | 1,019 (50.9%) | 896 (44.8%) |
| Missing | 44 (2.2%) | 42 (2.1%) |
| Psychological distress |  |  |
| No probable mental health issue | 1,641 (82.0%) | 1,628 (81.4%) |
| High Psychological Distress | 286 (14.3%) | 312 (15.6%) |
| Missing | 73 (3.6%) | 60 (3.0%) |
| Age |  |  |
| 65+ years | 315 (15.8%) | 456 (22.8%) |
| 55 - 64 years | 361 (18.1%) | 387 (19.4%) |
| 45 - 54 years | 392 (19.6%) | 382 (19.1%) |
| 35 - 44 years | 380 (19.0%) | 297 (14.9%) |
| 18 - 34 years | 551 (27.6%) | 476 (23.8%) |
| Missing | 1 (0.1%) | 2 (0.1%) |
| Gender |  |  |
| Male or Non-binary | 914 (45.7%) | 913 (45.6%) |
| Female | 1,084 (54.2%) | 1,085 (54.3%) |
| Missing | 2 (0.1%) | 2 (0.1%) |
| Disability |  |  |
| No | 1,500 (75.0%) | 1,481 (74.1%) |
| Yes | 405 (20.3%) | 408 (20.4%) |
| Missing | 95 (4.8%) | 111 (5.5%) |
| Income |  |  |
| $100,000 or more | 495 (24.8%) | 488 (24.4%) |
| Under $40,000 | 497 (24.9%) | 485 (24.3%) |
| $40,000 – $59,999 | 288 (14.4%) | 332 (16.6%) |
| $60,000 – $99,999 | 434 (21.7%) | 435 (21.8%) |
| Missing | 286 (14.3%) | 260 (13.0%) |
| Main activity in February & September 2020 | (February) | (September) |
| Employed | 1,154 (57.7%) | 1,069 (53.4%) |
| Unemployed | 367 (18.4%) | 374 (18.7%) |
| Retired | 319 (16.0%) | 456 (22.8%) |
| Other | 121 (6.0%) | 82 (4.1%) |
| Missing | 39 (1.9%) | 19 (0.9%) |
| Region |  |  |
| Other | 457 (22.9%) | 456 (22.8%) |
| Melbourne | 1,543 (77.1%) | 1,544 (77.2%) |
| Household Composition |  |  |
| Couple living alone | 548 (27.4%) | 589 (29.4%) |
| Person living alone | 397 (19.9%) | 444 (22.2%) |
| Couple with children | 646 (32.3%) | 599 (29.9%) |
| Single parent with children | 142 (7.1%) | 130 (6.5%) |
| Other | 214 (10.7%) | 200 (10.0%) |
| Missing | 53 (2.6%) | 38 (1.9%) |

^#^Unless otherwise stated. *Indicates statistically significant differences between the two lockdowns using an arbitrary p-value cut-off of 0.05 (37). Data consists of the participants who completed the survey at each time point; N= Number; SD=standard deviation.

Table S2: Baseline characteristics of the cohort with and without missing at the second lockdown

|  | Those who were not missing at the second lockdown | Those who were missing at the second lockdown |
| --- | --- | --- |
|  | N=1,008  N (%) or  mean (SD)^#^ | N=992  N (%) or  mean (SD)^#^ |
| Social Solidarity | 21.53 (4.36) | 21.12 (4.47) |
| Feeling connected with others |  |  |
| Agree | 408 (40.5%) | 377 (38.0%) |
| Mildly | 328 (32.5%) | 333 (33.6%) |
| Disagree | 221 (21.9%) | 214 (21.6%) |
| Missing | 51 (5.1%) | 68 (6.9%) |
| Staying connected with family and friends |  |  |
| Easy | 393 (39.0%) | 356 (35.9%) |
| Neither | 313 (31.1%) | 296 (29.8%) |
| Hard | 272 (27.0%) | 302 (30.4%) |
| Missing | 30 (3.0%) | 38 (3.8%) |
| Life Satisfaction |  |  |
| Low/Medium (Rated 0 to 6) | 463 (45.9%) | 474 (47.8%) |
| High (Rated 7 or 10) | 526 (52.2%) | 493 (49.7%) |
| Missing | 19 (1.9%) | 25 (2.5%) |
| ^*^Psychological distress |  |  |
| No probable mental health issue | 848 (84.1%) | 793 (79.9%) |
| High Psychological Distress | 124 (12.3%) | 162 (16.3%) |
| Missing | 36 (3.6%) | 37 (3.7%) |
| Age |  |  |
| 65+ years | 193 (19.2%) | 122 (12.3%) |
| 55 - 64 years | 234 (23.2%) | 127 (12.8%) |
| 45 - 54 years | 224 (22.2%) | 168 (17.0%) |
| 35 - 44 years | 179 (17.8%) | 201 (20.3%) |
| 18 - 34 years | 178 (17.7%) | 373 (37.7%) |
| Missing | 0 (0.0%) | 1 (0.1%) |
| Gender |  |  |
| Male or Non-binary | 439 (43.6%) | 475 (47.9%) |
| Female | 568 (56.3%) | 516 (52.0%) |
| Missing | 1 (0.1%) | 1 (0.1%) |
| Disability |  |  |
| No | 754 (74.8%) | 746 (75.2%) |
| Yes | 213 (21.1%) | 192 (19.4%) |
| Missing | 41 (4.1%) | 54 (5.4%) |
| Income |  |  |
| $100,000 or more | 263 (26.1%) | 232 (23.4%) |
| $60,000 – $99,999 | 215 (21.3%) | 219 (22.1%) |
| $40,000 – $59,999 | 147 (14.6%) | 141 (14.2%) |
| Under $40,000 | 245 (24.3%) | 252 (25.4%) |
| Missing | 138 (13.7%) | 148 (14.9%) |
| ^*^Main activity in February 2020 |  |  |
| Employed | 566 (56.2%) | 588 (59.3%) |
| Unemployed | 176 (17.5%) | 191 (19.3%) |
| Retired | 210 (20.8%) | 109 (11.0%) |
| Other | 40 (4.0%) | 81 (8.2%) |
| Missing | 16 (1.6%) | 23 (2.3%) |
| Region |  |  |
| Other | 233 (23.1%) | 224 (22.6%) |
| Melbourne | 775 (76.9%) | 768 (77.4%) |
| ^*^Household Composition |  |  |
| Couple living alone | 291 (28.9%) | 257 (25.9%) |
| Person living alone | 220 (21.8%) | 177 (17.8%) |
| Couple with children | 322 (31.9%) | 324 (32.7%) |
| Single parent with children | 66 (6.5%) | 76 (7.7%) |
| Other | 88 (8.7%) | 126 (12.7%) |
| Missing | 21 (2.1%) | 32 (3.2%) |

^#^Unless otherwise stated; *Indicates statistically significant differences between the groups using an arbitrary p-value cut-off of 0.05 (37). Data consists of the baseline participants who were or were not missing at the second lockdown survey; N= Number; SD=standard deviation.

Table S3: Results from univariable logistic regression modelling with psychological distress and life satisfaction as outcome at the first lockdown^**^

| Variable | Psychological Distress^#^  OR (95% CI), p-value | Life Satisfaction^##^  OR (95% CI), p-value | |
| --- | --- | --- | --- |
| ^*^Social Solidarity | 0.91 (0.89, 0.94), <0.001 | 1.13 (1.11, 1.16), <0.001 |  |
| ^*^Feeling connected with others  Agree |  |  |  |
| Mildly | 1.37 (0.97, 1.95), 0.07 | 0.46 (0.37, 0.58), <0.001 |  |
| Disagree | 5.03 (3.64, 6.96), <0.001 | 0.18 (0.14, 0.23), <0.001 |  |
| ^*^Staying connected with family and friends  Easy |  |  |  |
| Neither | 1.14 (0.80, 1.62), 0.46 | 0.58 (0.46, 0.72), <0.001 |  |
| Hard | 2.83 (2.08, 3.86), <0.001 | 0.34 (0.27, 0.43), <0.001 |  |
| ^*^Age  65+ years |  |  |  |
| 55 - 64 years | 1.91 (1.11, 3.31), 0.02 | 0.71 (0.52, 0.97), 0.03 |  |
| 45 - 54 years | 2.18 (1.28, 3.71), <0.001 | 0.71 (0.53, 0.97), 0.03 |  |
| 35 - 44 years | 2.43 (1.43, 4.12), <0.001 | 0.66 (0.48, 0.90), 0.01 |  |
| 18 - 34 years | 3.86 (2.37, 6.29), <0.001 | 0.62 (0.46, 0.82), <0.001 |  |
| Gender  Male or Non-binary |  |  |  |
| Female | 1.08 (0.84, 1.39), 0.54 | 0.89 (0.74, 1.06), 0.20 |  |
| ^*^Disability  No |  |  |  |
| Yes | 2.59 (1.96, 3.42), <0.001 | 0.48 (0.38, 0.60), <0.001 |  |
| ^*^Income  $100,000 or more |  |  |  |
| $60,000 - $99,999 | 1.71 (1.15, 2.54), 0.01 | 0.67 (0.51, 0.88), <0.001 |  |
| $40,000 - $59,999 | 1.63 (1.05, 2.53), 0.03 | 0.58 (0.43, 0.79), <0.001 |  |
| Under $40,000 | 2.01 (1.38, 2.94), <0.001 | 0.38 (0.29, 0.49), <0.001 |  |
| ^*^Main activity in Feb  Employed |  |  |  |
| Unemployed | 1.38 (1.01, 1.88), 0.04 | 0.50 (0.39, 0.64), <0.001 |  |
| Retired | 0.46 (0.29, 0.72), <0.001 | 1.00 (0.78, 1.29), 0.99 |  |
| Other | 1.26 (0.76, 2.10), 0.37 | 0.58 (0.39, 0.84), <0.001 |  |
| Region  Other |  |  |  |
| Melbourne | 1.32 (0.96, 1.81), 0.09 | 1.04 (0.84, 1.28), 0.71 |  |
| ^*^Household Composition  Couple living alone |  |  |  |
| Person living alone | 1.67 (1.15, 2.43), 0.01 | 0.57 (0.44, 0.75), <0.001 |  |
| Couple with children | 1.31 (0.93, 1.85), 0.13 | 0.86 (0.68, 1.08), 0.20 |  |
| Single parent with children | 1.67 (1.00, 2.77), 0.05 | 0.45 (0.31, 0.65), <0.001 |  |
| Other | 1.35 (0.85, 2.15), 0.21 | 0.47 (0.34, 0.65), <0.001 |  |

**Unadjusted models; OR= Odds Ratio; CI= Confidence Interval; Results using cross-sectional data are presented; **^#^**N_1_=1287; ^##^N_2_=1287; ^*^Statistically significant associations using an arbitrary p-value cut-off of 0.05 (37).

Table S4: Results from univariable logistic regression modelling with psychological distress and life satisfaction as outcome at the second lockdown^**^

| Variable | Psychological Distress^#^  OR (95% CI), p-value | Life Satisfaction^##^  OR (95% CI), p-value | |
| --- | --- | --- | --- |
| ^*^Social Solidarity | 0.89 (0.87, 0.92), <0.001 | 1.15 (1.12, 1.17), <0.001 |  |
| ^*^Feeling connected with others  Agree |  |  |  |
| Mildly | 0.97 (0.68, 1.39), 0.86 | 0.36 (0.29, 0.45), <0.001 |  |
| Disagree | 4.41 (3.22, 6.03), <0.001 | 0.12 (0.10, 0.16), <0.001 |  |
| ^*^Staying connected with family and friends  Easy |  |  |  |
| Neither | 0.74 (0.51, 1.08), 0.12 | 0.44 (0.35, 0.56), <0.001 |  |
| Hard | 1.80 (1.34, 2.42), <0.001 | 0.23 (0.18, 0.29), <0.001 |  |
| ^*^Age  65+ years |  |  |  |
| 55 - 64 years | 2.62 (1.64, 4.20), <0.001 | 0.75 (0.57, 0.98), 0.04 |  |
| 45 - 54 years | 2.83 (1.78, 4.51), <0.001 | 0.74 (0.56, 0.97), 0.03 |  |
| 35 - 44 years | 3.03 (1.86, 4.92), <0.001 | 0.60 (0.44, 0.81), <0.001 |  |
| 18 - 34 years | 5.04 (3.27, 7.75), <0.001 | 0.69 (0.53, 0.89), <0.001 |  |
| ^*^Gender  Male or Non-binary |  |  |  |
| Female | 1.13 (0.89, 1.44), 0.32 | 0.76 (0.64, 0.91), <0.001 |  |
| ^*^Disability  No |  |  |  |
| Yes | 1.94 (1.47, 2.55), <0.001 | 0.51 (0.40, 0.64), <0.001 |  |
| ^*^Income  $100,000 or more |  |  |  |
| $60,000 - $99,999 | 1.18 (0.81, 1.70), 0.39 | 0.67 (0.52, 0.88), <0.001 |  |
| $40,000 - $59,999 | 1.32 (0.90, 1.95), 0.16 | 0.70 (0.53, 0.93), 0.01 |  |
| Under $40,000 | 1.37 (0.96, 1.94), 0.08 | 0.53 (0.41, 0.68), <0.001 |  |
| ^*^Main activity in Sep  Employed |  |  |  |
| Unemployed | 1.64 (1.22, 2.21), <0.001 | 0.45 (0.35, 0.58), <0.001 |  |
| Retired | 0.52 (0.36, 0.75), <0.001 | 1.08 (0.87, 1.35), 0.47 |  |
| Other | 1.73 (1.01, 2.98), 0.05 | 0.68 (0.43, 1.08), 0.11 |  |
| ^*^Region  Other |  |  |  |
| Melbourne | 1.59 (1.16, 2.19), <0.001 | 0.85 (0.69, 1.06), 0.15 |  |
| ^*^Household Composition  Couple living alone |  |  |  |
| Person living alone | 1.63 (1.14, 2.33), 0.01 | 0.68 (0.53, 0.87), <0.001 |  |
| Couple with children | 1.62 (1.16, 2.26), <0.001 | 1.06 (0.84, 1.33), 0.62 |  |
| Single parent with children | 2.12 (1.29, 3.48), <0.001 | 0.46 (0.31, 0.69), <0.001 |  |
| Other | 1.76 (1.13, 2.74), 0.01 | 0.59 (0.43, 0.83), <0.001 |  |

**Unadjusted models; OR= Odds Ratio; CI= Confidence Interval; Results using cross-sectional data are presented; ^#^N_1_=1377; ^##^N_2_=1382; ^*^Statistically significant associations using an arbitrary p-value cut-off of 0.05 (37).

Table S5: Longitudinal associations of change in psychological distress and life satisfaction between the first and the second lockdowns^**^

| Variable | Psychological Distress^#^  OR (95% CI), p-value | Life Satisfaction^##^  OR (95% CI), p-value | |
| --- | --- | --- | --- |
| ^*^Social Solidarity at the first lockdown | 0.94 (0.89, 0.99), 0.02 | 1.07 (1.03, 1.12), <0.001 |  |
| ^*^Social Solidarity at the second lockdown | 0.93 (0.88, 0.97), <0.001 | 1.11 (1.07, 1.15), <0.001 |  |
| ^*^Feeling connected with others at the first lockdown  Agree |  |  |  |
| Mildly | 1.15 (0.67, 1.98), 0.62 | 0.68 (0.49, 0.94), 0.02 |  |
| Disagree | 2.49 (1.48, 4.19), <0.001 | 0.44 (0.29, 0.66), <0.001 |  |
| ^*^Feeling connected with others at the second lockdown  Agree |  |  |  |
| Mildly | 0.61 (0.32, 1.16), 0.13 | 0.40 (0.28, 0.58), <0.001 |  |
| Disagree | 3.53 (2.08, 5.99), <0.001 | 0.15 (0.10, 0.23), <0.001 |  |
| ^*^Staying connected with family and friends at the first lockdown  Easy |  |  |  |
| Neither | 0.92 (0.53, 1.60), 0.76 | 0.72 (0.51, 1.01), 0.06 |  |
| Hard | 1.48 (0.88, 2.49), 0.14 | 0.48 (0.33, 0.70), <0.001 |  |
| ^*^Staying connected with family and friends at the second lockdown  Easy |  |  |  |
| Neither | 1.03 (0.55, 1.94), 0.93 | 0.47 (0.31, 0.69), <0.001 |  |
| Hard | 1.71 (1.01, 2.91), 0.05 | 0.26 (0.18, 0.38), <0.001 |  |
| ^*^Age  65+ years |  |  |  |
| 55 - 64 years | 1.28 (0.59, 2.77), 0.53 | 1.08 (0.70, 1.68), 0.73 |  |
| 45 - 54 years | 1.99 (0.95, 4.15), 0.07 | 1.18 (0.76, 1.85), 0.46 |  |
| 35 - 44 years | 1.91 (0.88, 4.13), 0.10 | 0.89 (0.55, 1.44), 0.64 |  |
| 18 - 34 years | 2.65 (1.26, 5.58), 0.01 | 0.93 (0.58, 1.49), 0.75 |  |
| ^*^Gender  Male or Non-binary |  |  |  |
| Female | 1.08 (0.70, 1.65), 0.74 | 0.72 (0.54, 0.96), 0.03 |  |
| ^*^Disability  No |  |  |  |
| Yes | 0.98 (0.59, 1.64), 0.95 | 0.60 (0.41, 0.89), 0.01 |  |
| ^*^Income  $100,000 or more |  |  |  |
| $60,000 - $99,999 | 1.02 (0.55, 1.89), 0.95 | 0.73 (0.48, 1.10), 0.13 |  |
| $40,000 - $59,999 | 0.84 (0.42, 1.67), 0.62 | 0.71 (0.45, 1.12), 0.14 |  |
| Under $40,000 | 0.90 (0.49, 1.64), 0.73 | 0.66 (0.44, 1.00), 0.05 |  |
| ^*^Main activity in September  Employed |  |  |  |
| Unemployed | 1.51 (0.91, 2.52), 0.11 | 0.55 (0.37, 0.82), <0.001 |  |
| Retired | 0.81 (0.45, 1.45), 0.48 | 0.86 (0.60, 1.23), 0.41 |  |
| Other | 1.36 (0.42, 4.39), 0.60 | 0.50 (0.20, 1.23), 0.13 |  |
| Region  Other |  |  |  |
| Melbourne | 1.59 (0.93, 2.72), 0.09 | 0.81 (0.58, 1.14), 0.23 |  |
| ^*^Household Composition  Couple living alone |  |  |  |
| Person living alone | 0.76 (0.41, 1.41), 0.38 | 0.89 (0.59, 1.33), 0.56 |  |
| Couple with children | 1.13 (0.65, 1.97), 0.66 | 1.44 (1.00, 2.08), 0.05 |  |
| Single parent with children | 1.15 (0.46, 2.87), 0.77 | 0.96 (0.51, 1.80), 0.90 |  |
| Other | 0.80 (0.35, 1.83), 0.60 | 0.91 (0.52, 1.61), 0.75 |  |

**Unadjusted models; OR= Odds Ratio; CI= Confidence Interval; Results using cross-sectional data are presented; ^#^N_1_=591; ^##^N_2_=592; ^*^Statistically significant associations using an arbitrary p-value cut-off of 0.05 (37).
